# Phylogeography and cohesion species delimitation of California endemic trapdoor spiders within the *Aptostichus icenoglei* sibling species complex (Araneae: Mygalomorphae: Euctenizidae)

**DOI:** 10.1002/ece3.10025

**Published:** 2023-04-26

**Authors:** Lacie G. Newton, James Starrett, Emma E. Jochim, Jason E. Bond

**Affiliations:** ^1^ Department of Entomology & Nematology University of California Davis California USA

**Keywords:** Araneae, California Floristic Province, integrative species delimitation, speciation, ultraconserved elements

## Abstract

Species delimitation is an imperative first step toward understanding Earth's biodiversity, yet what constitutes a species and the relative importance of the various processes by which new species arise continue to be debatable. Species delimitation in spiders has traditionally used morphological characters; however, certain mygalomorph spiders exhibit morphological homogeneity despite long periods of population‐level isolation, absence of gene flow, and consequent high degrees of molecular divergence. Studies have shown strong geographic structuring and significant genetic divergence among several species complexes within the trapdoor spider genus *Aptostichus*, most of which are restricted to the California Floristic Province (CAFP) biodiversity hotspot. Specifically, the *Aptostichus icenoglei* complex, which comprises the three sibling species, *A*. *barackobamai*, *A*. *isabella*, and *A*. *icenoglei*, exhibits evidence of cryptic mitochondrial DNA diversity throughout their ranges in Northern, Central, and Southern California. Our study aimed to explicitly test species hypotheses within this assemblage by implementing a cohesion species‐based approach. We used genomic‐scale data (ultraconserved elements, UCEs) to first evaluate genetic exchangeability and then assessed ecological interchangeability of genetic lineages. Biogeographical analysis was used to assess the likelihood of dispersal versus vicariance events that may have influenced speciation pattern and process across the CAFP's complex geologic and topographic landscape. Considering the lack of congruence across data types and analyses, we take a more conservative approach by retaining species boundaries within *A*. *icenoglei*.

## Graphical abstract

Species delimitation in spiders has traditionally used morphological characters; however, certain mygalomorph spiders are heavily inclined toward morphological homogeneity despite long periods of population‐level isolation and consequent high degrees of molecular divergence. Our study aimed to explicitly test species hypotheses within the trapdoor spider *Aptostichus icenoglei* complex by implementing an integrative delimitation approach. We used genomic‐scale ultraconserved elements (UCEs) to evaluate genetic exchangeability and large‐scale ecological data to assess ecological interchangeability of genetic lineages in conjunction to determine cohesion species, with a subsequent biogeographical analysis to assess events that may have potentially influenced speciation pattern and process across the California Floristic Province's complex geologic and topographic landscape.

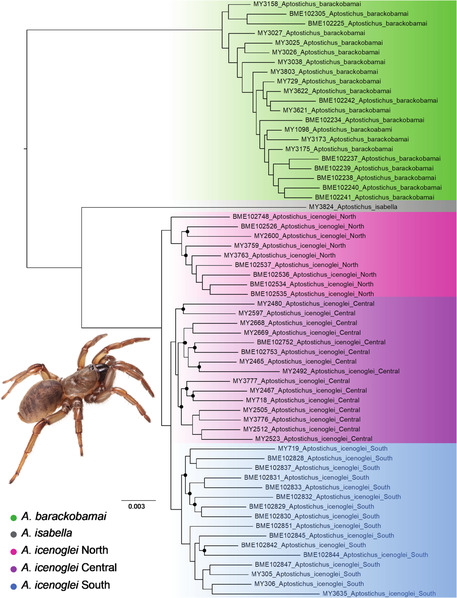

## 1 INTRODUCTION

The conceptual definition of what constitutes a species along with the relative importance of the varied processes by which new species arise continue to be much‐debated topics of discussion (de Queiroz, 2007; Hey, 2001; Wells et al., 2021). Species concepts typically emphasize disparate intrinsic biological properties (e.g., morphological differences, niche divergence, and genetic divergence) that can be differentially important with respect to species recognition and/or speciation process. Contingent factors, for example taxon characteristics and life history traits and point/stage in the speciation process, may render various concepts incompatible and/or delimit species in different ways; that is, one concept may recognize multiple distinct species whereas another may lump them together (de Queiroz, 2007).

Assessing species limits is particularly difficult in taxa with limited dispersal capabilities when reduced gene flow leads to high levels of population structuring. Taxa with high levels of genetic divergence and no gene flow can sometimes lead to speciation in the absence of notable morphological differentiation, essentially obscuring species boundaries. Specifically, non‐vagile taxa are closely tied to the landscape, such that as geological, topographical, and climatic changes occur over time, populations become geographically isolated with severely limited opportunity for gene flow (Bond et al., 2001; Derkarabetian et al., 2021; Starrett & Hedin, 2007; Weisrock & Larson, 2006). As these populations remain spatially isolated over relatively long periods of time and accumulate random mutations, genetic divergence builds through genetic drift and/or natural selection for adaptive alleles in population(s) that inhabit newly available niche space. When spatial isolation is coupled with occupation of new niche space, one would expect each population to not only exhibit genetic divergence but also morphological, behavioral, and/or physiological differences (Freudenstein et al., 2016). However, if genetically diverged populations remain stationary in niche space (i.e., niche conservatism; Wiens & Graham, 2005), then it would be plausible for morphological stasis to occur in the absence of differing selective pressures, resulting in genetic lineages that are morphologically indistinguishable (Bond et al., 2001; Cerca et al., 2021; Mas‐Peinado et al., 2018). In that case, it is likely that species diversity will be underestimated because traditional approaches that primarily apply morphological distinctiveness are more commonly used in species delimitation (Bond et al., 2021). Thus, implementing a species concept focusing on one biological property/data type could potentially misrepresent the actual number of species present if that property was not important in the speciation process (Abbott et al., 2013; de Queiroz, 2007).

The species concept applied in a given system has implications for downstream delimitation decisions and thus must be explicitly stated in any species delimitation study. Nevertheless, in many taxonomic studies (e.g., in spider taxonomy), an explicit species concept is seldom stated (Bond et al., 2021). A species concept that focuses strictly on morphological differentiation has the potential to overlook cryptic species that may otherwise be genetically diverged to the point that genomic incompatibilities preclude gene flow (Barroso et al., 2010; Battey & Klicka, 2017; Holland et al., 2004; Weisrock & Larson, 2006). Alternatively, molecular approaches to species delimitation have been shown to overestimate species diversity when local population structuring is viewed as “species divergence”. Specifically, single‐locus approaches such as DNA barcoding along with GMYC, as well as multiple‐locus approaches (e.g., multispecies coalescent methods) that assume panmixia are prone to identifying population structuring as opposed to speciation events (Hamilton et al., 2014; Hedin et al., 2015; Sukumaran & Knowles, 2017). In such systems, localized divergence of neutral alleles may be inconsequential when populations come into secondary contact, so any species delimitation approach that relies primarily on genetic differentiation has the potential to overestimate species diversity when applying these methods. Consequently, a species concept that incorporates multiple biological properties as an integrative species delimitation approach that weighs evidence from multiple independent sources is likely to more accurately identify true evolutionary species diversity.

The Cohesion Species Concept (CSC; Templeton, 1989) has arguably already solved the problems of too little versus too much gene flow and provides the hypothetical and conceptual foundation for framing integrative species delimitation. The CSC posits that a cohesion species must constitute an independently evolving evolutionary lineage and must be genetically exchangeable and/or ecologically interchangeable (Templeton, 1989). Specifically, a primary tenet of a cohesion species is that it comprises populations that exchange genes and occupy similar niche space. This concept can be applied to essentially all taxa, integrates multiple biological properties that are potentially important in the speciation process, and provides a methodological framework in which species hypotheses can be tested (Barraclough, 2019; Bond & Stockman, 2008; Templeton, 1989; Wells et al., 2021). Thus, it is particularly useful when evaluating species boundaries in morphologically homogenous taxa prone to cryptic diversity in conjunction with a high amount of population structuring at small spatial scales (Bond & Stockman, 2008; Hendrixson et al., 2013, 2015; Newton et al., 2020).

In this paper, we will apply the CSC to a species delimitation problem in a previously characterized lineage of trapdoor spiders in the genus *Aptostichus* Simon (Araneae: Mygalomorphae: Euctenizidae), specifically species in the *Aptostichus icenoglei* sibling species complex. Mygalomorph spiders are notorious for being morphologically static relative to the other, more diverse spider groups placed in the sister infraorder Araneomorphae (Opatova et al., 2019); they have relatively long lifespans and limited dispersal capabilities which makes their populations more vulnerable to genetic structuring at small spatial scales (Bond & Stockman, 2008; Cooper et al., 2011; Hamilton et al., 2014; Harvey et al., 2018; Starrett & Hedin, 2007), thus underscoring the interplay of genetic versus ecological interchangeability when evaluating divergence at the species/population interface in these highly structured taxa. The questions we pose are related first to genetic exchangeability—do these populations constitute distinct genetic lineages, and if so, are they ecologically interchangeable, or not? If these genetically distinct lineages are ecologically interchangeable, then the unavoidably subjective question arises of how heavily one weights genetic divergence versus ecological/adaptive divergence, or the lack thereof (discussed below).

The *Aptostichus icenoglei* sibling species complex comprises three species: *A*. *icenoglei* Bond, *A*. *barackobamai* Bond, and *A*. *isabella* Bond. Like other *Aptostichus* species, they construct thin wafer trapdoors from silk and the surrounding substrate; they are geographically widespread throughout three regions in the California Floristic Province (CAFP), a known biodiversity hotspot (Bond, 2012; Myers et al., 2000). The CAFP has a complex geological, climatic, and topographical history, which has highly influenced the speciation pattern and process of many plants (Anacker et al., 2011; Baldwin et al., 2011; Cole et al., 2011; Eckert et al., 2008; Grivet et al., 2006; Kraft et al., 2010; Liston et al., 2007; Rundel, 2011) and animals (Alexander & Burns, 2006; Chatzimanolis & Caterino, 2007; Leaché et al., 2009; Oliver & Shapiro, 2007; Pardikes et al., 2017; Rios & Álvarez‐Castañeda, 2010; Sgariglia & Burns, 2003; Spinks & Shaffer, 2005; Vandergast et al., 2006), especially non‐vagile taxa such as salamanders (Jockusch et al., 2020; Martínez‐Solano et al., 2007; Wake, 1997), harvestmen (Emata & Hedin, 2016), scorpions (Bryson et al., 2016), and mygalomorph spiders (Bond & Stockman, 2008; Bond, 2012; Hedin et al., 2013; Leavitt et al., 2015; Satler et al., 2011). Dispersal‐limited taxa have proven to be particularly useful in broadening our understanding of the historical biogeography of the CAFP (Emata & Hedin, 2016; Hedin et al., 2013; Martínez‐Solano et al., 2007). Evolutionary divergence, influenced by barriers to dispersal either because of biotic (e.g., competition or predation) or abiotic factors (e.g., geologic, geographic, or environmental factors), can be detected at both relatively small spatial and timescales for these low‐dispersal taxa (Hedin et al., 2013). The combination of dispersal‐limited taxa generally being relatively morphologically homogenous yet having significant genetic divergence suggests the primary mode of divergence would be influenced by vicariance events, such as geological activity creating barriers to gene flow, as opposed to adaptive divergence (e.g., niche divergence through competition). Thus, evidence for biogeographical barriers remains intact in these systems for longer time periods and can potentially reveal multiple barriers to dispersal (i.e., both long‐term and short‐term barriers; Hedin et al., 2013; Martínez‐Solano et al., 2007), so one can expect patterns seen in genetic variation of low‐dispersal organisms to closely reflect the geological history of the region in which they are distributed.

*Aptostichus barackobamai* and *A*. *icenoglei* are relatively widespread and exhibit evidence of cryptic diversity (i.e., morphologically similar yet found in a variety of habitats across a sizable geographic range) found in other mygalomorph groups (Hamilton et al., 2014; Hendrixson et al., 2013; Hendrixson & Bond, 2005; Starrett et al., 2018; Starrett & Hedin, 2007) as well as other *Aptostichus* species (Bond et al., 2001; Bond & Stockman, 2008). *Aptostichus isabella,* on the contrary, is only known from one specimen collected near Lake Isabella in the southern Sierran foothills. *Aptostichus icenoglei* is distributed throughout the Transverse Ranges from the Los Angeles Basin to the Santa Ana/San Jacinto Mountains as well as the mountains and hills surrounding San Diego (Bond, 2012). The primary habitat types for *A*. *icenoglei* include coastal chaparral forest and coastal range open woodland shrub and coniferous forest (Bond, 2012). *Aptostichus barackobamai* is found in primarily mixed redwood and coniferous forests in the northern Coastal Ranges as well as along the northern rim of the Central Valley, with one population in the Sutter Buttes (Bond, 2012). Altogether, these likely represent a diversity of habitat types spread across a number of different California ecoregions. Mitochondrial data from Bond (2012) indicate considerable population genetic structuring, especially in *A*. *icenoglei*, which is likely influenced by the typical mygalomorph life history traits discussed above. This, in conjunction with notable molecular divergence as well as a diversity of habitats, suggests that *A*. *barackobamai* and *A*. *icenoglei* populations, respectively, have been isolated from gene flow for an extended period of time, which would increase speciation potential (i.e., both likely comprise more than one species; Barraclough, 2019).

The primary objective of this study was to use multiple lines of evidence, specifically morphological, ecological, and genomic‐scale data (i.e., ultraconserved elements, UCEs; Faircloth et al., 2012) and to evaluate phylogenetic relationships, species boundaries, and historical biogeography within the *A*. *icenoglei* complex. We explicitly tested species hypotheses within this assemblage by implementing a CSC‐based approach. We first evaluated genetic exchangeability using clustering analyses to assess the potential for gene flow and then assessed ecological interchangeability of genetic lineages with a niche‐based distribution modeling approach. Additionally, biogeographic analysis was used to investigate the likelihood of dispersal versus vicariant events that may have influenced speciation pattern and process across the CAFP's complex topographic and geologic landscape.

## 2 METHODS

### 2.1 Taxon sampling

**FIGURE 1:**
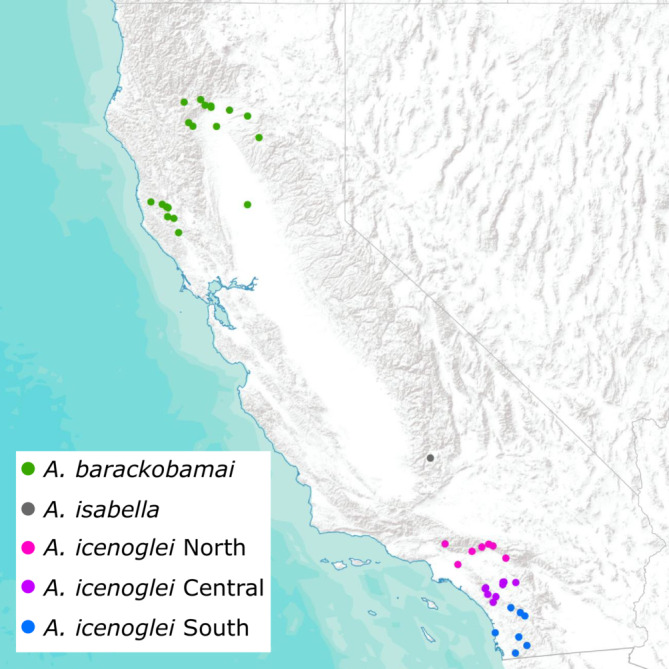
Geographic distributions of *Aptostichus icenoglei* sibling species complex lineages. Inset legend denotes color scheme for each of the lineages recovered in Figure 2.

We sampled 62 individuals overall for the three species within the complex using both specimens from Bond (2012) and new records (Figure 1; see Table S1 for locality information). *Aptostichus barackobamai* was collected across its geographic range in northern California for a total of 21 samples, and *A*. *icenoglei* was collected throughout its range in southern California for a total of 40 samples. Only one specimen of *A*. *isabella* was included in this study due to collecting constraints (i.e., only one individual of this species has ever been collected and a burrow has not yet been found containing this species; Bond, 2012).

### 2.2 Sequence capture

Data for ultraconserved elements were produced following the methods described in Faircloth et al. (2012) with subsequent modifications in Starrett et al. (2017), Hedin et al. (2018), and Kulkarni et al. (2020). We extracted genomic DNA from leg tissue for *A*. *barackobamai* and *A*. *icenoglei* individuals using the Blood and Tissue DNeasy kit (Qiagen) following the manufacturer's protocol. The lone *A*. *isabella* individual, a museum voucher kept in 80% ethanol and stored at room temperature, had DNA extracted from leg tissue following the “MMYT protocol” from Tin et al. (2014) with modifications in Derkarabetian et al. (2019). DNA quantification and quality check were performed using Qubit 3.0 Fluorometer (Life Technologies) and agarose gel, respectively. Excluding *A*. *isabella*, 250 ng of DNA was sonicated into fragments ranging from 200 to 1000 bp using an ultrasonicator (Covaris E220). UCE libraries were generated with the KAPA Hyperprep Kit (Roche) with universal adapters and iTru5/7 barcodes (Glenn et al., 2019; BadDNA@UGA) with slight modifications on a few steps for *A*. *isabella* (for details see Derkarabetian et al., 2019). Libraries were hybridized at 60°C for 24 h to the Spider probe set (Kulkarni et al., 2020) following the version 4 chemistry protocol (Arbor Biosciences). Hybridization‐enriched library pools were sequenced with 150 bp paired‐end reads on the HiSeq4K at the University of California Davis DNA Technologies Core. Additional individuals were sent to Rapid Genomics (Florida) for library preparation and sequencing.

Sequence processing and analyses were performed on the Farm Community HPC at the University of California, Davis. Reads were filtered and trimmed using Illumiprocessor (Faircloth, 2013) and Trimmomatic (Bolger et al., 2014) in the Phyluce 1.7.1 pipeline (Faircloth, 2015). De novo assemblies with the cleaned paired‐end and single‐end reads were performed using SPAdes v. 3.14.1 with the isolate option (Prjibelski et al., 2020). Scaffolds were matched with 65% identity and 65% coverage to the modified probe list from Maddison et al. (2020), which is a blend of the Arachnid (Faircloth, 2017; Starrett et al., 2017) and Spider (Kulkarni et al., 2020) probe sets. MAFFT (Katoh & Standley, 2013) was used to align individual locus datasets, and alignments with locus occupancy (i.e., completeness) minimums of 50%, 75%, and 90% were obtained. Alignment masking was performed with TrimAl v.1.2 (Capella‐Gutierrez et al., 2009) using default settings.

SNP datasets were generated for *A*. *icenoglei* only with Phyluce from the 50%, 75%, and 90% minimum occupancy loci. Reads were mapped against corresponding scaffolds with BWA (Li & Durbin, 2009), implemented in Phyluce, and phased alignments were generated for each minimum locus completeness set. Phased alignments were screened for SNPs, with five sets of single random SNP per locus generated to test for SNP set sensitivity.

### 2.3 Phylogenetic & biogeographic analyses

**FIGURE 2:**
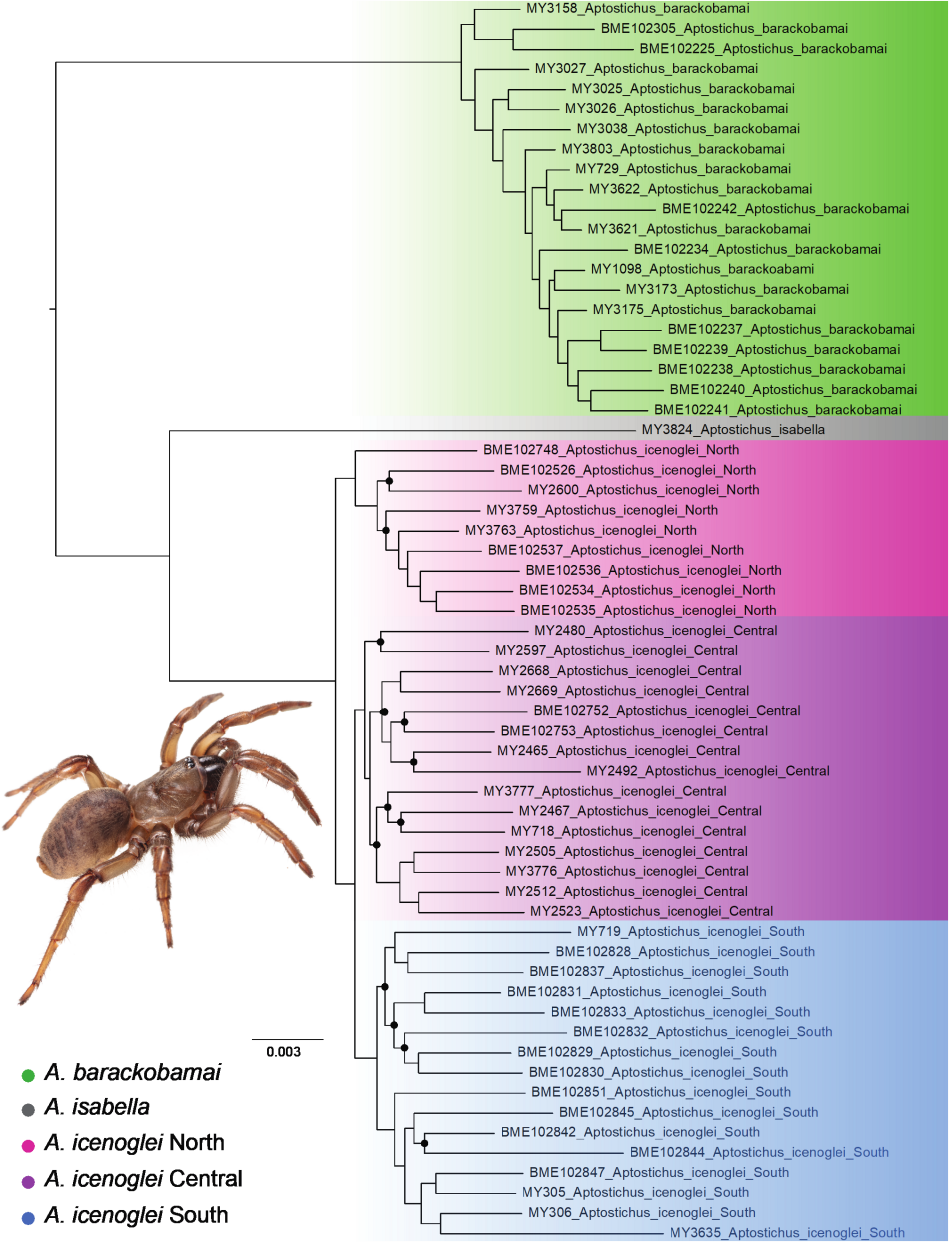
Maximum likelihood tree inference based on the 90p data set. Nodes with Bootstrap values having support <90 are denoted by black dots. Spider inset is *Aptostichus icenoglei* from San Bernardino Co. (*A*. *icenoglei* Central).

Phylogenies were estimated for three different data sets (50, 75, and 90 percent locus completeness; Figure 2 and Figures S1 and S2) with a maximum likelihood inference using IQ‐TREE v2.1.2 (Minh et al., 2020). Model selection was performed by ModelFinder (Kalyaanamoorthy et al., 2017), which is implemented in IQ‐TREE, and support values were inferred from 1000 replicates of ultrafast bootstrapping (Hoang et al., 2018). Our phylogenies were visualized in FigTree v1.4.1 with midpoint rooting (midpoint rooting produces a result consistent in other analyses; Bond, 2012) and compared to assess congruence among clades. We also conducted two coalescent‐based analyses for the 75p and 90p data sets. Gene trees for each locus were constructed using RAxML v8.0.12 for each data set and used to generate a coalescent‐based tree with ASTRAL‐III (Zhang et al., 2018). Multispecies coalescent (MSC) bootstrapping was run with ASTRAL v.5.7.4 and 100 pseudoreplicates (Simmons et al., 2019). For downstream analyses, we employed the ML phylogeny based on the largest amount of taxon coverage and with the most robust support values (i.e., the phylogeny with 90 percent minimum locus completeness).

**FIGURE 3:**
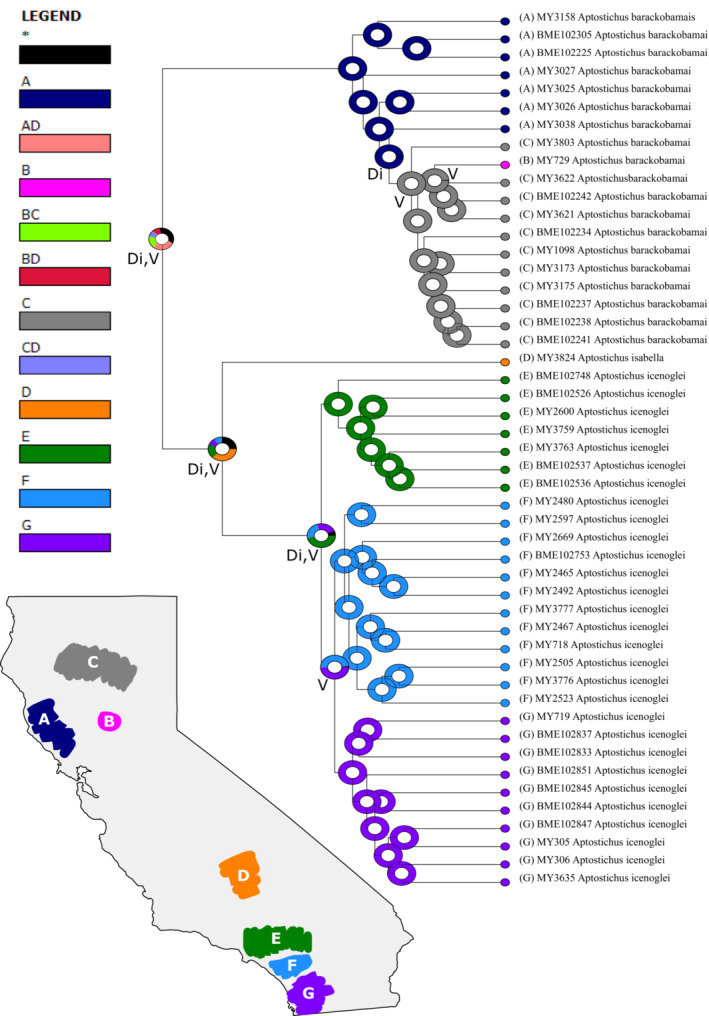
Ancestral area distribution estimation was inferred with DIVALIKE+j analysis implemented in RASP. Terminals and inferred ancestral distributions coloration corresponds to the assigned geographic regions (A = lower San Diego county; B = upper San Diego county/Santa Ana Mtns; C = Transverse Mtn Ranges; D = southern Sierras; E = northern rim of Central Valley; F = Sutter Buttes; and G = Northern Coast Ranges) as depicted on the map (bottom left) or combination of regions (i.e., AD, BC, BD, and CD) as depicted in the legend (top left). Biogeographic events are marked on the nodes: Di = dispersal; V = vicariance.

Biogeographic analyses were generated using Reconstruct Ancestral State in Phylogenies (RASP; Yu et al., 2015) with dispersal constraints (i.e., dispersal multipliers set to 0.01 for adjacent areas and 0.0001 for non‐adjacent areas) to account for their limited dispersal capacity and using our 90p consensus tree from IQ‐TREE. Model testing was conducted using the R package BioGeoBEARS (Matzke, 2014), implemented in RASP, and the best‐fit model was chosen based on the weighted AICc scores (Figure 3). The distribution range of this complex was divided into seven areas: (A) lower San Diego county; (B) upper San Diego county/Santa Ana Mtns; (C) Transverse Ranges (San Gabriel & San Bernardino Mtns); (D) southern Sierras; (E) northern rim of Central Valley; (F) Sutter Buttes; and (G) Northern Coast Ranges.

### 2.4 Cohesion species delimitation

To assess species boundaries within *A*. *barackobamai* and *A*. *icenoglei*, we employed the methodological framework for delimiting cohesion species from Bond and Stockman (2008) that evaluates two cohesion mechanisms: genetic exchangeability and ecological interchangeability. We used our 90p topology as the baseline evolutionary framework for establishing the “basal starting point” to identify potential separately evolving lineages (for details see flowchart in Bond & Stockman, 2008) within *A*. *icenoglei* and *A*. *barackobamai*. Due to the paraphyletic grade of lineages with respect to geography (i.e., northern Coast Range species did not form a clade) within *A*. *barackobamai*, we designated all the individuals as part of one evolving lineage that was not tested further for genetic and ecological exchangeability. For *A*. *icenoglei*, we also used our topology from the MSC tree resampling (Figure S6) as additional guidance for establishing lineage designations, which resulted in 3 lineages: North, Central, and South (see Figure 2). We evaluated the distributions of these lineages as well as performed morphological and genetic clustering analyses to assess the potential for gene flow. Genetic exchangeability was rejected if any allopatric lineage forms an apparently separate clustering pattern from other lineages, or if any parapatric lineage has a separate clustering pattern *and* an obvious barrier to gene flow.

For morphological data, we quantified 25 continuous character measurements for 30 males (10 males from each lineage; Table S2). All measurements were recorded in millimeters and were quantified with a Leica M165C stereomicroscope using the Leica Application Suite software and a digital camera. Measurements were transformed to log‐normal values, and a principal component analysis was conducted using the *prcomp* function in the R package stats (R Core Team, 2022) and visualized in ggplot2 (Wickham, 2016), following Hamilton et al. (2016).

**FIGURE 4:**
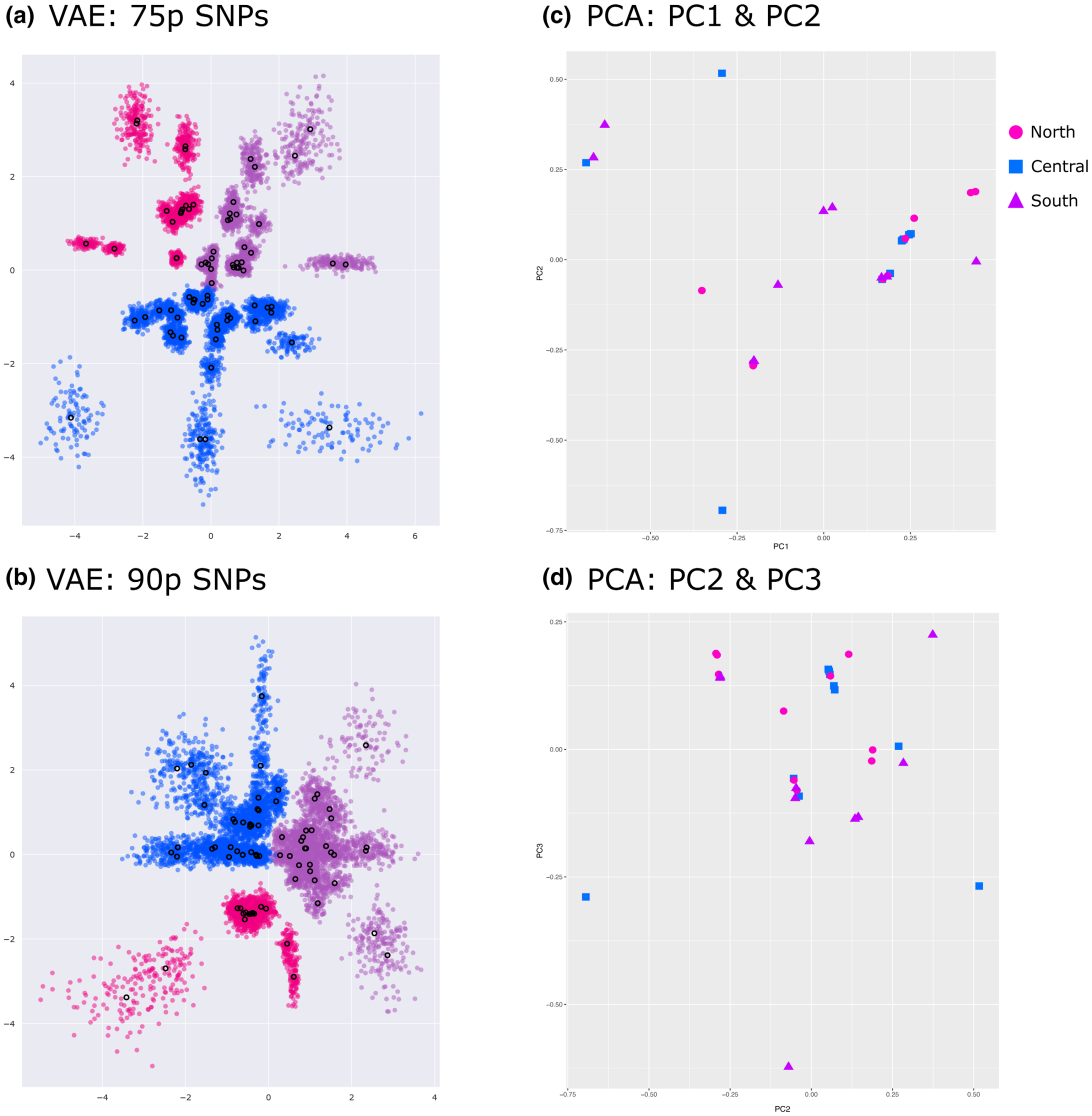
Clustering analysis plots of *Aptostichus icenoglei* lineages for both molecular and morphological data sets. Same color scheme for each lineage as previous figures, with PCA plots also having distinguishing symbols for each lineage as seen in the top right legend. (a) VAE plot constructed from the 75p SNP data set. (b) VAE plot constructed from the 90p SNP data set. (c) PCA plot, with PC1 and PC2, constructed from morphological measurements. (d) PCA plot, with PC2 and PC3, constructed from morphological measurements.

We conducted two genetic clustering analyses. Variational AutoEncoder (VAE), an unsupervised machine learning approach derived from Bayesian probability theory, was used to visualize clustering of these lineages (Figure 4; for details see Derkarabetian et al., 2019). This class of neural networks takes large‐scale SNP data as input and compresses this high‐dimensional data through several encoding layers into two‐dimensional latent variables, which is subsequently reconstructed by uncompressing the latent variables through several decoding layers (Derkarabetian et al., 2019). SNP datasets were converted to one‐hot encoding, which converts categorical data into numerical data as needed for certain machine learning algorithms and used as input for VAE analyses. Three replicates per random SNP set were conducted for each dataset (15 total replicates per dataset), and the replicate for each dataset with the least amount of loss during decoding was used for visualization (Figure 4). CLADES, a supervised machine learning approach, was used to further test species hypotheses (Pei et al., 2018). A 90% minimum locus completeness data set with all *A*. *icenoglei* individuals was the input data for CLADES. The delimitation analysis was performed using a training model of genetic characteristics of species generated from a short‐range endemic arachnid genus (*Metanonychus*) that has similar natural history characteristics to mygalomorph spiders (Metano_CLADES model from Derkarabetian et al., 2022).

**FIGURE 5:**
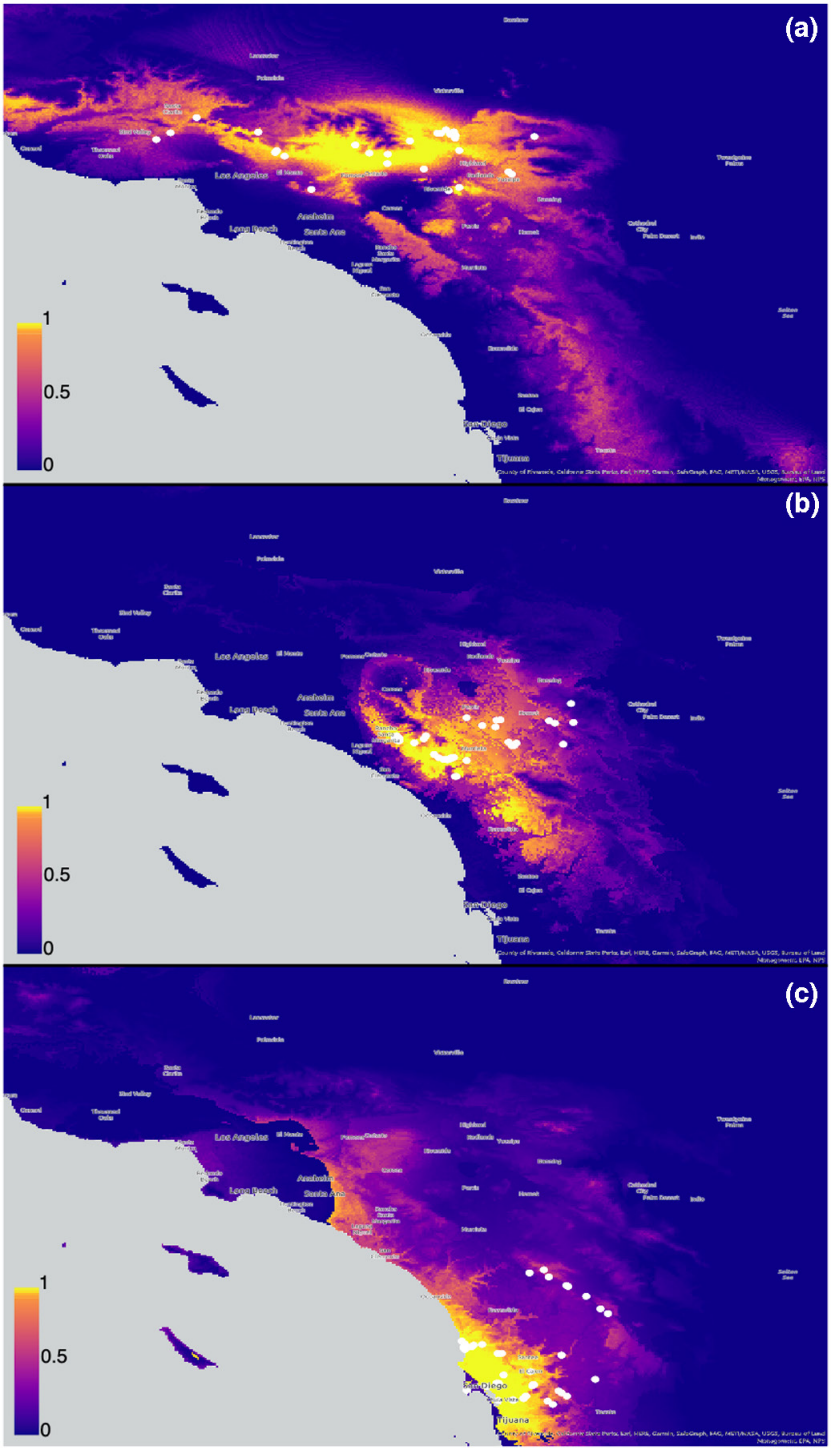
Species distribution models for each of the *Aptostichus icenoglei* lineages. (a) *A*. *icenoglei* North lineage. (b) *A*. *icenoglei* Central lineage. (c) *A*. *icenoglei* South lineage. Legend insets denote probability of occurrence with cooler colors indicating a lower probability; warmer colors (yellow/orange) indicate a higher probability.

Niche‐based species distribution modeling (SDMs) with measures of SDM overlaps for each lineage were used as a proxy for ecological interchangeability, with ecological interchangeability rejected if both the niche equivalency and niche similarity tests are more different than expected by chance (i.e., niche divergence). Current climate data for 19 bioclimatic variables averaged from 1970 to 2000 were downloaded from WorldClim version 2.1 at 30 arc‐second spatial resolution (https://www.worldclim.org/data/worldclim21.html; Fick & Hijmans, 2017). Climate layers were cropped to encompass the geographic area of interest and converted to a raster stack using R packages raster (Hijmans, 2015) and rgdal (Bivand et al., 2019). Highly correlated variables with a Pearson correlation coefficient > .80, estimated using the R package ENMTools (Warren et al., 2021), were removed. The remaining bioclimatic variables (see Table S3) were used in conjunction with occurrence records from the current study as well as records from Bond (2012) that could confidently be assigned to a lineage to generate SDMs, with duplicate records deleted prior to SDM construction. The R package ENMeval (Kass et al., 2021) was used to estimate the SDM for each lineage by implementing MaxEnt (Phillips & Dudík, 2008), which is a machine learning program that uses a maximum entropy algorithm. Multiple points within a 30 arc‐second grid cell were removed (i.e., only retaining one record per grid cell) by ENMeval during the modeling step to reduce potential for spatial autocorrelation. To limit the likelihood of overfitting while also accounting for goodness of fit, multiple feature classes and regularization multipliers were chosen to generate a total of 30 models (see Tables S4–S7 for model parameters and stats). Model selection was based on AICc, with the best model having a delta AICc of zero and was subsequently used in downstream analyses (Figure 5).

Statistical comparisons of SDMs for each sister lineage comparison were conducted with niche overlap, niche equivalency, and niche similarity tests in ENMTools (Warren et al., 2008, 2010). We used the Schoener's *D* statistic (Schoener, 1968) to calculate the niche overlap for each lineage comparison, which ranges from 0 (no overlap) to 1 (complete overlap). We carried out two tests, niche equivalency and niche similarity, to evaluate the significance of niche overlap with a randomization procedure (Warren et al., 2008, 2010). The niche equivalency test, a one‐tailed test, assesses whether the two niches being compared are identical or not. If the observed niche overlap value is significantly lower than the null distribution of randomized *D* values, then the niches are not identical (i.e., not equivalent; Figure S7). Considering the limitation of relying only on occurrence records for the niche equivalency test (Warren et al., 2008), we also employed the niche similarity test, a two‐tailed test, to assess whether niche overlap between lineages relative to the niche spaces available to those lineages is more similar or different than expected by chance (niche conservatism or niche divergence, respectively; Figures S8 and S9). We estimated three potential background regions for each lineage in ArcGIS Pro v2.8 (ESRI): (1) minimum area polygons based on occurrence points; (2) minimum area polygons based on SDM raster grid cells with a habitat suitability score threshold greater than 0.5 (i.e., a polygon was generated around every grid cell with a habitat suitability score greater than 0.5); and (3) minimum area polygons based on SDM raster grid cells with a habitat suitability score threshold greater than 0.75 (see Figures S10–S12 for reference).

## 3 RESULTS

### 3.1 UCE stats

The UCE data are summarized in Table 1. Across all individuals, contigs that matched to the probes had a mean length of 1010 bp, with an average of 545 contigs over 1 kb per individual. After aligning, filtering, and trimming these UCE contigs, we had three data matrices with varying minimum locus completeness percentages: 50p containing 1336 loci with 1,101,054 total bp, 75p containing 835 loci with 692,091 total bp, and 90p containing 242 loci with 204,134 total bp. For each *A*. *icenoglei* SNP data set, there were 1120 SNPs, 668 SNPs, and 195 SNPS for the 50p, 75p, and 90p respectively.

**TABLE 1:** UCE stats.

Species	Sample ID	Total raw reads	Total cleaned reads	Total contigs	Total Base pairs	Mean contig length	95 CI length	Min contig length	Max contig length	Median length	Contigs >1 kb
*Aptostichus barackobamai*	BME102225	4,420,078	7,830,491	1332	1,384,323	1039.3	11.7	229	7453	1018	708
*Aptostichus barackobamai*	BME102234	3,812,115	6,628,564	1303	1,380,869	1059.8	12.4	230	6012	1034	716
*Aptostichus barackobamai*	BME102237	3,330,201	5,663,593	1344	1,517,872	1129.4	14.1	230	7288	1074.5	827
*Aptostichus barackobamai*	BME102238	3,707,668	6,442,682	1302	1,267,103	973.2	10.4	155	5966	959.5	576
*Aptostichus barackobamai*	BME102239	3,510,383	6,056,168	1306	1,262,668	966.8	10.4	218	6109	962	571
*Aptostichus barackobamai*	BME102240	2,715,448	4,722,596	1321	1,304,052	987.2	10.8	185	6847	963	591
*Aptostichus barackobamai*	BME102241	3,388,498	5,867,580	1354	1,429,568	1055.8	11.6	241	4637	1029	733
*Aptostichus barackobamai*	BME102242	4,821,353	8,457,382	1120	1,122,821	1002.5	14.4	231	8145	989	540
*Aptostichus barackobamai*	BME102305	4,304,076	7,602,546	984	843,236	856.9	9.5	230	2887	860.5	296
*Aptostichus barackobamai*	MY1098	2,415,859	4,686,229	1104	1,216,927	1102.3	9.3	230	2835	1137.5	744
*Aptostichus barackobamai*	MY3025	4,268,144	7,912,442	1197	1,225,139	1023.5	9.4	234	3456	1042	665
*Aptostichus barackobamai*	MY3026	3,350,390	6,578,386	1074	1,241,439	1155.9	10.4	179	3269	1171.5	759
*Aptostichus barackobamai*	MY3027	3,451,363	6,744,182	1151	1,554,991	1351	14.5	242	6358	1352	926
*Aptostichus barackobamai*	MY3038	4,215,949	8,233,007	1170	1,506,863	1287.9	12.4	210	5002	1304	924
*Aptostichus barackobamai*	MY3158	4,358,520	8,520,364	1119	1,487,339	1329.2	13.3	204	3869	1344	897
*Aptostichus barackobamai*	MY3173	4,188,958	8,180,139	1151	1,680,454	1460	15.3	219	7753	1470	989
*Aptostichus barackobamai*	MY3175	4,111,218	8,027,550	1148	1,707,380	1487.3	15.5	240	6611	1509.5	989
*Aptostichus barackobamai*	MY3621	4,191,434	8,196,361	1274	1,893,179	1486	12.8	230	4614	1512	1128
*Aptostichus barackobamai*	MY3622	4,413,976	8,629,470	1236	1,827,553	1478.6	12.9	231	4498	1500	1089
*Aptostichus barackobamai*	MY3803	4,948,423	9,700,833	1207	1,538,978	1275	11.7	239	5061	1278	950
*Aptostichus barackobamai*	MY729	2,838,864	5,535,308	1200	1,857,610	1548	15.2	229	7389	1580	1073
*Aptostichus isabella*	MY3824	255,356	410,418	351	112,875	321.6	5.4	229	1077	290	2
*Aptostichus icenoglei*	BME102535	3,018,802	5,908,597	1053	1,186,312	1126.6	14.5	230	7522	1115	678
*Aptostichus icenoglei*	BME102536	2,465,496	4,822,054	1274	1,543,663	1211.7	12.2	230	6235	1186.5	941
*Aptostichus icenoglei*	BME102537	3,745,511	7,327,284	1100	1,101,546	1001.4	9.2	235	2537	1022	588
*Aptostichus icenoglei*	BME102748	1,992,694	3,855,530	1188	1,037,676	873.5	8.8	233	4290	863	328
*Aptostichus icenoglei*	BME102752	2,776,732	5,422,034	1114	1,094,827	982.8	8.6	231	2344	1000	558
*Aptostichus icenoglei*	BME102753	2,776,732	4,417,498	1195	1,159,099	970	9.1	229	3599	986	573
*Aptostichus icenoglei*	BME102828	2,316,700	4,487,134	1193	991,958	831.5	8.7	191	3267	831	247
*Aptostichus icenoglei*	BME102829	2,417,689	4,665,418	943	810,743	859.7	9.7	227	3042	867	262
*Aptostichus icenoglei*	BME102830	2,128,137	4,113,040	1051	909,845	865.7	10.3	193	3852	858	295
*Aptostichus icenoglei*	BME102831	2,671,417	5,155,788	1013	779,982	770	9	154	5540	761	156
*Aptostichus icenoglei*	BME102832	1,759,619	3,408,090	1221	899,874	737	7.1	191	3176	732	105
*Aptostichus icenoglei*	BME102833	2,212,181	4,279,630	1157	1,024,709	885.7	8.8	229	3204	881	347
*Aptostichus icenoglei*	BME102837	2,299,688	4,411,190	1019	906,935	890	9.5	230	2278	888	347
*Aptostichus icenoglei*	BME102842	1,975,434	3,812,588	1037	818,933	789.7	8.8	238	5750	788	196
*Aptostichus icenoglei*	BME102844	1,964,343	3,796,994	1191	952,279	799.6	9.7	232	4227	782	237
*Aptostichus icenoglei*	BME102845	1,958,630	3,777,170	972	882,606	908	10.4	229	3826	902.5	346
*Aptostichus icenoglei*	BME102847	2,073,595	4,004,664	1175	1,067,725	908.7	9.4	237	3031	894	396
*Aptostichus icenoglei*	BME102851	1,966,455	3,811,742	1028	841,342	818.4	11.2	232	7274	795.5	205
*Aptostichus icenoglei*	MY2465	10,149,464	9,232,542	990	886,950	895.9	10.2	178	1929	886.5	376
*Aptostichus icenoglei*	MY2467	9,805,774	9,008,121	1013	902,588	891	10.3	168	2792	888	370
*Aptostichus icenoglei*	MY2480	5,172,058	9,587,452	1073	1,126,520	1049.9	15.1	230	8634	1032	574
*Aptostichus icenoglei*	MY2492	4,440,918	8,243,968	1104	1,085,531	983.3	12	225	4132	967	508
*Aptostichus icenoglei*	MY2505	4,614,117	8,499,404	1098	1,110,392	1011.3	10.7	173	3993	1015.5	582
*Aptostichus icenoglei*	MY2512	4,058,619	7,461,057	995	874,528	878.9	10.2	185	2798	865	349
*Aptostichus icenoglei*	MY2523	4,530,508	8,315,839	1044	1,113,337	1066.4	12.6	226	3665	1045	594
*Aptostichus icenoglei*	MY2597	4,530,508	9,193,740	1087	1,189,932	1094.7	11.9	236	3758	1101	654
*Aptostichus icenoglei*	MY2600	4,885,983	8,991,862	1184	1,298,298	1096.5	11	180	3905	1100	743
*Aptostichus icenoglei*	MY2668	4,259,227	7,854,572	1028	964,622	938.3	10.1	218	2885	938.5	433
*Aptostichus icenoglei*	MY2669	4,744,563	8,670,518	1110	1,250,851	1126.9	13.7	230	5716	1126.5	731
*Aptostichus icenoglei*	MY305	4,060,054	7,482,832	1055	901,193	854.2	9.1	205	2228	858	328
*Aptostichus icenoglei*	MY306	4,437,521	8,162,959	1080	1,025,976	950	10.3	185	4356	951.5	467
*Aptostichus icenoglei*	MY3635	2,311,741	4,303,471	1237	1,024,072	827.9	9	232	3849	829	264
*Aptostichus icenoglei*	MY3759	4,785,470	9,033,324	1031	814,409	789.9	8.4	119	1627	790	241
*Aptostichus icenoglei*	MY3763	4,658,003	8,736,611	1099	926,173	842.7	8.7	183	2113	849	341
*Aptostichus icenoglei*	MY3776	5,701,942	10,709,261	1031	829,842	804.9	8.4	220	1708	815	264
*Aptostichus icenoglei*	MY3777	5,578,622	10,517,313	1012	828,068	818.2	8.8	230	2579	824	272
*Aptostichus icenoglei*	MY718	5,235,415	9,565,653	1020	1,054,146	1033.5	13.1	187	6305	1013	529
*Aptostichus icenoglei*	MY719	3,890,591	7,047,193	1061	1,087,100	1024.6	11.1	186	2717	1026	572

### 3.2 Phylogenetic and biogeographic analyses

All estimated phylogenies fully supported (i.e., 100 for IQ‐TREE or one for ASTRAL analyses) species level divergence among the three previously delineated morphological species within this sibling complex (see Figure 2 and Figures S1–S6). Also, all *A*. *icenoglei* lineage divergences (North, Central, and South) were highly, if not fully, supported across the majority of phylogenetic trees (i.e., all IQ‐TREE and ASTRAL analyses); however, despite recovering North and South lineages as monophyletic and highly supported (i.e., >90) in both the 75p and 90p MSC bootstrapping analyses, there was uncertainty in placement of several Central lineage individuals with both analyses (Figures S5 and S6).

RASP analysis (Figure 3) inferred an unresolved ancestral range for the ancestor of the complex, with only .1968 probability of a relatively widespread ancestor along the southern Sierras and in the Transverse Ranges that then dispersed to the north with a vicariance event, splitting the ancestor of *A*. *barackobamai* from the ancestor of *A*. *isabella* + *A*. *icenoglei*. Within *A*. *barackobamai*, there were dispersal events northeastward along the northern Coast Ranges to the northern rim of the Central Valley/Sierra Nevada, and vicariance events splitting the northern Coast Ranges populations from northeastern populations as well as the Sutter Buttes population from the northern Sierra Nevada population. The split between the ancestor of *A*. *isabella* and the ancestor of *A*. *icenoglei*, with a .25 probability, was potentially the result of dispersal further south and a vicariance event. For the *A*. *icenoglei* lineages, the most likely scenario involved dispersal to the south toward the Peninsular Ranges with vicariance splitting the North from Central+South lineages and subsequent vicariance splitting Central from South.

### 3.3 Cohesion species delimitation

Table 2 summarizes results for each genetic exchangeability analysis. Geographic distribution assessments for each lineage comparison were considered parapatric. One comparison had no obvious barrier to gene flow (i.e., Central and South lineages), whereas the other comparison of North and Central+South lineages had an unlikely chance of gene flow occurring due to the LA Basin acting as a geographic barrier (see Figure 1). Three clustering analyses, one with morphological data and two with molecular data, were also used to inform the possibility of gene flow. PCA analysis of the quantitative morphological measurements reveal no distinct clustering for any of the lineages (Figure 4). Similarly, the CLADES analysis with the Metano_CLADES training model identified one species. In contrast, the VAE analysis for 50p indicates three very distinct clusters corresponding to each lineage for both the mean and standard deviation (Figure S13); however, although VAE analyses for 75p and 90p show separation between the lineages for the mean, there is a small amount of overlap for the standard deviation between Central and South lineages (Figure 4).

**TABLE 2:** Summary of *Aptostichus icenoglei* cohesion species delimitation assessment.

Lineage comparison	Genetic exchangeability				
	Geographical barrier	PCA (morphology)	VAE (molecules)	CLADES (molecules)	Conclusion
Central to South	Parapatric, no obvious barrier	Significant overlap	Small overlap of clusters	1 species	Fail to reject GE
North to Central + South	Parapatric, potential barrier (LA Basin)	Significant overlap	Separate clusters	1 species	Reject GE
	**Ecological interchangeability**				
	**N**_**a**_ **, N**_**b**_	**Niche overlap value**	**Niche equivalency test**	**Niche similarity test**	**Conclusion**
Central to South	42, 55	0.4595	*p* < .05	*p* < .025, niche conservatism	Fail to reject EI
North to Central + South	29, 97	0.3873	*p* < .05	*p* < .025, niche conservatism	Fail to reject EI

*Note*: N_a_ and N_b_ values are the number of occurrence records for the first and second lineages used in a comparison, respectively. The niche similarity test background region is based on the raster polygons where only grid cells with habitat suitability scores >0.75 were retained.

Table 2 summarizes results for each ecological interchangeability analysis. Niche equivalency was rejected for both lineage comparisons, indicating that their niches are not identical (Figure S7). Niche similarity test results were different depending on the background region selected. Central occurrence points compared with the South background, determined by a minimum bounding polygon connecting its occurrence points, were not significantly different; however, the reciprocal comparison of South occurrence points to Central background was significantly more similar than expected by chance (i.e., niche conservatism; Figure S8). Central occurrences compared with the South background, determined by minimum bounding polygons based on raster grid cells with either habitat suitability scores >0.5 or >0.75, and vice versa indicated niche conservatism (i.e., more similar than expected compared with the null distribution; Figure S8). When comparing the Central+South occurrence records to the minimum bounding polygon connecting occurrence points defining the background region of North, the results show no significant difference; yet, the reciprocal comparison is significantly more similar than expected (Figure S9). All comparisons of North versus Central+South and vice versa suggest niche conservatism when background regions are defined by minimum bounding polygons based on raster grid cells with either habitat suitability scores >0.5 or >0.75 (Figure S9).

## 4 DISCUSSION

Prior taxonomic work on the *A*. *icenoglei* assemblage identified three species based on morphological distinctiveness; however, there was molecular (i.e., mitochondrial) evidence that the two geographically widespread species (*A*. *icenoglei* and *A*. *barackobamai*) could include additional cryptic diversity (Bond, 2012). Our study used a cohesion species‐based delimitation approach from Bond and Stockman (2008) to expand on the evaluation of species boundaries within the complex. Once evolutionary lineages were delineated, based on the topology and high support values (i.e., >0.95) from both the 90p IQ‐TREE and the 90p MSC bootstrapping (Figure 2 and Figure S6), we recognized three distinct lineages within the nominal species *A*. *icenoglei*: North, Central, and South. In contrast, the paraphyletic grade with respect to geography within *A*. *barackobamai* leads us to retain the current species boundaries at this time (i.e., *A*. *barackobamai* populations comprise a single species owing to their apparent genetic exchangeability). Although it is possible that sampling more populations is warranted, particularly where a modest‐sized gap exists between the Coast Range populations and northern Central Valley rim/Sierra populations (Figure 1), intensive sampling efforts in parts of the Mendocino National Forest did not yield additional populations. Failing to initially reject the null hypothesis that *A*. *barackobamai* comprises a single lineage, we limit our targeted assessment of genetic and ecological exchangeability to *A*. *iceonoglei* lineages. Specifically, we utilized morphological measurements, genomic‐scale SNP data, and niche‐based distribution modeling to evaluate and test cohesion species boundaries within *A*. *icenoglei*; as we discuss in detail below, these analyses produced conflicting results, inferring one to three species. The unsupervised machine learning (VAE) analysis with the 50p dataset and niche equivalency tests are consistent with the three species hypothesis (i.e., North, Central, and South lineages are all separate cohesion species). The two species hypothesis (i.e., North and Central+South lineages are cohesion species) is supported by a known geographic barrier to gene flow (i.e., LA Basin) and VAE analyses with both 75p and 90p datasets. In contrast, the morphological data, supervised machine learning (CLADES) analysis, and niche similarity tests support what is essentially the null hypothesis that *A*. *icenoglei* lineages all comprise one single species. When considering all lines of evidence, limitations of datasets and analyses, and mygalomorph life history characteristics we retain the current species delimitation of *A*. *icenoglei* as one cohesion species (discussed further below).

### 4.1 Speciation and phylogeography

Spiders in the *A*. *icenoglei* complex, like most mygalomorphs, have limited dispersal capabilities and relatively long generation times (Bond, 2012; Harvey et al., 2018; Hedin et al., 2013; Hendrixson et al., 2013), which contributes to their tendency to have population structure at relatively small spatial scales. The molecular data show genetic divergence across the *A*. *icenoglei* complex and within *A*. *icenoglei* populations, thus populations have likely been isolated from gene flow for a long period of time, indicating the increased potential for speciation (Barraclough, 2019). The three nominal species (*A*. *barackobamai*, *A*. *isabella*, and *A*. *icenoglei*) are distributed across different regions of the CAFP and have been delimited based on distinct morphological differences in secondary mating structures (clasper morphology; Bond, 2012), providing evidence that gene flow has not occurred between them for a long period of time. Within *A*. *icenoglei* lineages, our VAE analyses with 75p and 90p datasets support the lack of gene flow between the North and Central+South lineages over an extended time period.

All species within the complex, with potentially the exception of *A*. *isabella*, seem to have similar microhabitat requirements (e.g., north‐facing shady slopes) despite their occurrence in different ecoregions, similar to other mygalomorph taxa in the CAFP (Hedin et al., 2013; Hedin & Carlson, 2011; Leavitt et al., 2015). Our niche similarity tests show evidence of niche conservation within *A*. *icenoglei* lineages, with the caveat that various analyses yielded different results when minimum bounding polygons versus raster grid cell thresholds parameters were considered. Many studies have used the niche similarity test to evaluate overlap in niche space (Hendrixson et al., 2013; McCormack et al., 2009; Newton et al., 2020; Starrett et al., 2018; Warren et al., 2008), yet very few are explicit about the background region they chose to incorporate into the analysis (McCormack et al., 2009; Newton et al., 2020; Starrett et al., 2018). In addition, as far as we are aware, no other study other than Newton et al. (2020) has explicitly tested multiple background regions to evaluate the impact background region choice has on the analysis. Our background region choices for the current study were chosen based on previous studies (minimum bounding polygon; McCormack et al., 2009; Starrett et al., 2018) and polygons reflecting raster grid cells with habitat suitability score thresholds (> 0.5 and > 0.75) that better reflect the suitable habitat space available (i.e., not including large gaps of uninhabitable areas that are included in the minimum bounding polygon). The minimum bounding polygon yielded conflicting results for both North versus Central+South comparisons and Central versus South comparisons, which is most likely attributed to the aforementioned uninhabitable areas included in the analysis that potentially obscures a signal of niche conservatism. Although we attempted to incorporate a more biologically realistic background region, it is possible that our habitat suitability thresholds slightly inflated the inference of niche conservatism and thus may warrant additional future testing and evaluation.

Niche conservatism, in conjunction with restricted gene flow, suggests that speciation scenarios in which vicariant events separate populations with subsequent reproductive isolation through genetic drift, as opposed to ecological differentiation, may apply across the *A*. *icenoglei* complex. This pattern is also supported by our biogeographic analysis, but caution should be used when interpreting these results considering our ultrametric tree was not dated and, thus, cannot pinpoint with certainty the geological/climatic events that potentially influenced these splits. First, a vicariant event (after range expansion; Figure 4) is inferred for the split of the ancestor of *A*. *barackobamai* and ancestor of *A*. *isabella + A*. *icenoglei*. This phylogeographic break potentially coincides with uplift of the Transverse Ranges approximately 5 mya (Norris & Webb, 1990), which likely cut off the potential for gene flow, and has been hypothesized for other CAFP taxa (Alexander & Burns, 2006; Calsbeek et al., 2003; Feldman & Spicer, 2006; Reilly et al., 2015; Rissler et al., 2006). Second, the split between *A*. *isabella* and *A*. *icenoglei* possibly occurred due to vicariance. This split could be attributed to the Tehachapi Mountains acting as a barrier to dispersal, which has also been inferred for other taxa (Calsbeek et al., 2003; Chatzimanolis & Caterino, 2007; Rissler et al., 2006). Third, vicariance was inferred for the split between the North lineage and Central+South lineages, which could be associated with periodic inundations of the LA Basin (Jacobs et al., 2004) that might have resulted in habitat fragmentation, also hypothesized for the mahogany Jerusalem cricket (*Stenopelmatus* “mahogani”; Vandergast et al., 2006) and stream‐dwelling frogs (*Pseudacris cadaverina*; Phillipsen & Metcalf, 2009).

### 4.2 Species limits within *Aptostichus icenoglei* and taxonomic implications

Although our integrative approach considered multiple independent lines of evidence, our conflicting results circle back to the unavoidably subjective question of how much weight should be given to genetic divergence versus morphological/ecological divergence (or lack thereof) when delimiting species with extreme population structuring. Should we elevate genetically diverged lineages to species status despite the lack of observed morphological/ecological differences? One could argue that identifying and describing cryptic diversity can be important not only for more accurate biodiversity measures but also conservation management plans (i.e., evolutionary significant units; Ryder, 1986). For example, Fennessy et al. (2016) delimited four species of giraffe based on a genetic isolation criterion and placed special emphasis on conservation management of the northern giraffe *Giraffa camelopardalis* and its four recognized subspecies due to the severity of population declines when compared to other related species. In our case, the North lineage has been severely threatened by fires over the last 20 years compared with Central+South lineages. Specifically, more than half of the North lineage population occurrence records fall within a fire perimeter that occurred between 2000 and 2020, compared with approximately 20 percent for Central+South occurrence records falling within a fire perimeter. Failing to recognize the obvious genetic diversity in the North lineage could result in its loss due to lack of a management plan targeting their distribution in the Transverse Ranges or trying to manage all of *A*. *icenoglei* as one species could also result in not having adequate recognition and consequently protection for the North lineage.

Alternatively, one could argue that there is no practical value of recognizing genetically diverged lineages as separate species considering the lack of any visible diagnostic character/difference in ecological role (Freudenstein et al., 2016). Specifically, Freudenstein et al. (2016) argued that possessing both a unique ecological role and phenotypic differences are imperative when recognizing distinct species units. However, even this argument is rife with subjectivity; for example, how much phenotypic difference is enough to distinguish lineages as separate species? Also, it has been established that the speciation process is a continuum in which certain biological properties can be affected at different points along that continuum (Abbott et al., 2013; de Queiroz, 2007). Thus, it is feasible for geographically separated populations to accumulate enough genetic divergence for reproductive isolation despite still having morphological and ecological stasis. However, if one was to view species only in the context of a small snapshot in time (i.e., time‐limited view of species; Freudenstein et al., 2016) and assumes reproductive isolation based only on genetic divergence, then that raises the question of what happens if/when secondary contact occurs with a sibling sister “species” or lineage (i.e., time‐extended view of species; Freudenstein et al., 2016). One of the two options is possible if secondary contact occurs: (1) morphological and/or ecological differences could emerge to maintain reproductive isolation (reinforcement), or (2) hybridization occurs and genetic divergence between populations is eliminated via the effects of gene flow. Freudenstein et al. (2016) argue that viewing species as ecologically distinctive with historical gene flow combines both the temporal and phenotypic natures of species and alleviates the ambiguity of whether or not genetically diverged yet morphologically/ecologically homogenous lineages will remain diverged in the future. Thus, the most conservative taxonomic approach would be to require rejection of *both* genetic and ecological interchangeability for identifying separate cohesion species.

Studies spanning different animal taxa that have utilized CSC‐based delimitation approaches have highlighted the importance of evidence for adaptive divergence when delimiting species (Bond & Stockman, 2008; Leaché et al., 2009; Newton et al., 2020; Rengifo‐Correa et al., 2021). For mygalomorphs, Bond and Stockman (2008), the study upon which our CSC framework is based, delimited *A*. *miwok* and *A*. *stephencolberti* within the *A*. *atomarius* species complex based on mitochondrial data plus evidence of adaptive divergence (i.e., coastal dune habitats and lighter abdominal coloration). In a follow‐up study, Garrison et al. (2020) found evidence of chemosensory‐associated gene families under selection in dune endemics compared with their inland sister lineages, further elucidating patterns of ecological differentiation between coastal and inland sister species. Another example within mygalomorphs includes Newton et al. (2020) who initially identified five genetically distinct lineages within the *Antrodiaetus unicolor* species complex; however, genetic and ecological exchangeability assessments led to the delimitation of three species, not five, based on molecular, behavioral, and morphological data. In a similar study, Leaché et al. (2009) identified five phylogeographic groups within the coast horned lizard *Phyrnosoma coronatum* species complex based on molecular data, yet an assessment of climatic niche models and morphometrics of cranial horn shapes led to the delimitation of three species based on multiple operational criteria. Lastly, another example involves the difficult taxonomic status of kissing bugs within the *Triatoma phyllosoma* species group, where species limits have been hard to establish given occurrences of hybridization and cryptic diversity (Rengifo‐Correa et al., 2021). Despite relatively low genetic divergence and the potential for hybridization, species within the *T*. *phyllosoma* complex can be distinguished based on morphological characters (i.e., head phenotype) and are all considered separate cohesion species.

Our analytical results separately inferred one to three species within *A*. *icenoglei* depending on the dataset and analysis used, but the final decision, arguably subjective, comes down to emphasizing mygalomorph life history characteristics and acknowledging limitations for each data type/analysis (discussed further below). The three species hypothesis was dismissed due to: (1) the less conservative niche equivalency test (Warren et al., 2008), (2) the possibility that the 50 percent locus completeness SNP dataset overly inflated cluster separation between Central + South, and (3) no obvious barrier to gene flow between Central and South lineages. The two species hypothesis is not substantiated based on morphological and ecological similarity between lineages, yet it is supported by rejecting genetic exchangeability as inferred by the VAE cluster separation with higher/more conservative locus completeness percentage datasets (75p and 90p) and a probable hard barrier to gene flow, the LA Basin, between North and Central+South. Although the LA Basin is likely impeding gene flow due to urban development and habitat fragmentation, the small likelihood of a potential corridor of habitat suitable for dispersal along the northern Basin rim/southeastern San Bernardino mountains cannot be completely dismissed (e.g., figure 1 in Vandergast et al., 2006). The one species hypothesis is supported by morphological and ecological data as well as an implementation of a supervised machine learning analysis on the 90p SNP dataset. Notably, the CLADES training model used in our study is potentially not appropriate for mygalomorphs, and the prevalence of morphological homogeneity (Bond & Stockman, 2008; Harvey et al., 2018; Hedin et al., 2013; Leavitt et al., 2015; Newton et al., 2020) and ecological similarity (Cooper et al., 2011; Hedin & Carlson, 2011; Rix et al., 2020) among mygalomorphs could obscure actual evolutionary diversity. The flowchart in Bond and Stockman (2008) suggests that rejecting genetic exchangeability for parapatric lineages, but not rejecting ecological interchangeability, can still potentially indicate separate cohesion species if niche conservatism is occurring. However, this view must be balanced with acknowledging that sparse, if any, evidence for adaptive divergence could indicate that reproductive isolation is not complete (i.e., ecological divergence is usually correlated with reproductive isolation; Freudenstein et al., 2016; Rissler & Apodaca, 2007), especially for parapatric lineages that still have the potential for gene flow in the future. Considering the lack of congruence across data types and analyses, we are taking the most conservative approach by retaining species boundaries within *A*. *icenoglei* until additional data types, both ecological and whole genomes, can be included for evaluating cohesion species identity.

### 4.3 Limitations of analyses and future prospects

We believe that the supervised machine learning analysis has limitations due to potential shortcomings with the training data set devised using unrelated taxa. Although we see great value in attempting to establish a training dataset integrating biologically/ecologically relevant characteristics, it is difficult to assess how applicable this dataset can be to other dispersal‐limited taxa, especially across different taxonomic orders and biogeographical regions (Derkarabetian et al., 2022). First, the taxon *Metanonychus*, on which the training dataset was established, diverged approximately 25 mya, whereas the *A*. *icenoglei* sibling species complex likely diverged much later, which could artificially conflate deeper divergences with a predetermined “species cutoff” value, even if shallower species divergences are observed. Second, *Metanonychus* is found throughout the Pacific Northwest (Derkarabetian et al., 2019) whereas the *A*. *icenoglei* complex is found throughout the California Floristic Province, a biodiversity hotspot characterized by the intense complexity of geological, climatic, and topographic changes (Myers et al., 2000). One could argue that the overall complexity of the CAFP might influence the speciation process of low dispersal taxa in a different manner from how topographic changes in the Pacific Northwest would to the point that the genetic signatures may manifest differently. Specifically, as there are more topographical changes (both in number and intensity), the more chances there are for speciation through vicariance when compared to fewer/less drastic topography shifts (Badgley et al., 2017).

Our VAE analysis with the lower locus completeness dataset (50p) showed obvious separation between all three of the *A*. *icenoglei* lineages, whereas our higher locus completeness datasets (75p and 90p) retained only enough signal to maintain the North lineage as a separate cluster but not for Central or South lineages (Figure 4). VAE relies on the inherent structure present in the data (Derkarabetian et al., 2019), and previous studies have shown that VAE analyses have been heavily influenced by the filtering parameters for the SNP datasets (Martin et al., 2021; Newton et al., 2020). Specifically, if a lower threshold for locus completeness is allowed in a dataset the more likely it is to “over‐split”, whereas more stringent filtering (i.e., a high threshold for locus completeness) can remove potentially important signal and “under‐split” the amount of diversity. Because our higher locus completeness datasets retained the same clustering patterns, we are confident that they accurately reflect genetic divergence, and that the 50p dataset separation pattern for Central and South is an artifact of the filtering choice. Thus, it is important to be mindful of the potential filtering strategies for these SNP datasets, and best practices if utilizing VAE as a species delimitation method would be to use multiple filtering strategies to identify possible data artifacts versus actual structure.

There are known caveats for using niche‐based distribution modeling approaches as a proxy for evaluating ecological interchangeability. First, it has to be acknowledged that large‐scale ecological data, which are based on a very small time frame of 30 years (i.e., 1970–2000), used for building the SDMs potentially lacks the resolution needed for detecting very small‐scale habitat differences which may be important for detecting adaptive divergence (Massatti & Knowles, 2014; Newton et al., 2020; Starrett et al., 2018). The microhabitat preferences for these spiders, which includes shaded ravines, north‐facing slopes, and specific soil types (Bond, 2012), found within the heterogeneous landscapes throughout the CAFP are potentially not identified in the SDMs by even the best resolution available. Thus, our niche similarity tests using these models likely do not detect the potential for microhabitat niche divergence and consequently suggest the need for studies that assess fine‐scale data on variables like temperature, precipitation, burrow features (e.g., size and depth), and soil composition.

Second, as discussed above, background region choice can heavily impact the results of niche similarity tests, thus incorporating multiple regions with biologically relevant constraints may provide a more rigorous application. Third, considering that our proxy of ecological interchangeability was only based on the abiotic factors contributing to niche space (i.e., bioclimatic variables and occurrence records to build an SDM), one could argue that there were other potential avenues of ecological divergence that could have been included in this study for a more robust evaluation of ecological interchangeability. There are potential biotic factors (e.g., competition with other taxa, difference in prey items across microhabitats, or non‐overlapping breeding periods) that could distinguish lineages from one another. For example, other studies delimiting mygalomorph species have included behavioral traits when applicable (e.g., non‐overlapping breeding periods; Hendrixson et al., 2015; Hendrixson & Bond, 2005; Prentice, 1997). Unfortunately, the lack of available natural history data for many fossorial mygalomorphs (Bond, 2012; Hedin et al., 2013; Starrett et al., 2017) have limited use of this type of data in species delimitation decisions.

Given these limitations, there are many potential avenues in which researchers can begin to bridge these gaps in knowledge. First, generating more datasets comprising low‐dispersal taxa with varying divergence times and across other biogeographical regions that can be used to train models for supervised machine learning methods such as CLADES, will likely aid the robustness of this approach (Derkarabetian et al., 2022). Second, accumulating more natural history data for mygalomorphs will not only provide valuable general ecological information but may also be used as additional evidence in species delimitation. For example, pitfall trapping spiders in areas where occurrence records of each species/lineage of interest is well‐known to collect specimens can be informative for both breeding period times and gut content analysis to identify prey items that are being ingested (i.e., can inform potential for ecological divergence). Third, the advent of assembled and annotated genomes for non‐model taxa, specifically in *Aptostichus*, will likely pave the way toward utilizing these data not only for reconstructing evolutionary relationships but also identifying genes that contribute to potential adaptive divergence across the landscape (Johnson, 2018).

Overall, our study emphasized the efficacy of implementing a cohesion species‐based delimitation approach across all taxa, but especially for assessing the potential of cryptic diversity. Using genome‐wide UCEs in conjunction with morphological and ecological data to evaluate genetic and ecological exchangeability provided multiple independent lines of evidence that covered multiple biological properties potentially important in the speciation process. Specifically, this integrative approach underscored how different data types or approaches alone could either over‐ or under‐split diversity estimates, yet taking them all into consideration led to a more robust species delimitation hypothesis within the *A*. *icenoglei* complex. Typically, such studies of taxa with extreme population structuring favor recognizing cryptic species, whereas herein, we have shown that an integrative approach, considering multiple lines of evidence, has the capacity to retain (lump) populations as a single species. Moreover, we reinforce the capability of the Cohesion Species Concept in providing both the conceptual and experimental framework for conducting such tests. Finally, our biogeographic analysis reveals that vicariance likely played a dominant role in the speciation process across the entire complex, further highlighting the impact of the complex geological, climatic, and topographical changes across the CAFP on speciation process.

## AUTHOR CONTRIBUTIONS

**Lacie G. Newton:** Conceptualization (equal); data curation (equal); formal analysis (lead); funding acquisition (supporting); investigation (lead); methodology (equal); visualization (equal); writing – original draft (lead); writing – review and editing (equal). **James Starrett:** Conceptualization (supporting); data curation (equal); formal analysis (supporting); funding acquisition (supporting); investigation (supporting); methodology (equal); supervision (equal); visualization (supporting); writing – review and editing (equal). **Emma E. Jochim:** Data curation (supporting); formal analysis (supporting); visualization (supporting); writing – review and editing (equal). **Jason E. Bond:** Conceptualization (equal); data curation (equal); formal analysis (supporting); funding acquisition (lead); investigation (supporting); project administration (equal); resources (lead); supervision (lead); visualization (equal); writing – original draft (supporting); writing – review and editing (equal).

## CONFLICT OF INTEREST STATEMENT

Collectively, the authors (LGN, JS, EEJ, JEB) have no conflict of interest to disclose.

## Supporting information


Appendix S1.

## Data Availability

All sequence data can be found in Sequence Read Archive with the BioProject ID PRJNA949729. All tree files, data matrices, and VAE one‐hot encoded files can be found here: https://doi.org/10.25338/B8MD2Z. All scripts used in this study can be found here: https://github.com/lgnewton/AptIceClade_SpDelim.
