# Supplementary material for: Phylogeography and cohesion species delimitation of California endemic trapdoor spiders within the Aptostichus icenoglei sibling species complex (Araneae: Mygalomorphae: Euctenizidae)
Source: Ecol Evol. 2023 Apr 26;13(4):e10025. doi: 10.1002/ece3.10025 (PMC10133383; doi:10.1002/ece3.10025)
Supplement: Supplementary file 1 — Appendix S1. [file ECE3-13-e10025-s001.docx]

**Supplemental Tables**

Supplemental Table 1. Locality information for all samples used in the study.

| **Specimen ID** | **Specific Epithet** | **Locality Details** | **Latitude** | **Longitude** | **Country** | **State** | **County** | **Collection Date** | **Collectors** |
| --- | --- | --- | --- | --- | --- | --- | --- | --- | --- |
| BME102225 | *Aptostichus barackobamai* | CA-Hwy 253, E of Boonville | 39.0547 | -123.245 | United States | California | Sonoma | 1/16/2021 | L. Newton, E. Joachim |
| BME102234 | *Aptostichus barackobamai* | CA-Hwy 36, S of Platina | 40.3177 | -122.7822 | United States | California | Shasta | 2/3/2021 | L. Newton |
| BME102237 | *Aptostichus barackobamai* | Trinity Mtn Rd | 40.6851 | -122.6395 | United States | California | Shasta | 2/4/2021 | L. Newton |
| BME102238 | *Aptostichus barackobamai* | S Shore Dr, Whiskeytown Lake | 40.6081 | -122.5586 | United States | California | Shasta | 2/4/2021 | L. Newton |
| BME102239 | *Aptostichus barackobamai* | Middle Creek Rd | 40.5955 | -122.4528 | United States | California | Shasta | 2/4/2021 | L. Newton |
| BME102240 | *Aptostichus barackobamai* | Middle Creek Rd | 40.5955 | -122.4528 | United States | California | Shasta | 2/4/2021 | L. Newton |
| BME102241 | *Aptostichus barackobamai* | Middle Creek Rd | 40.5955 | -122.4528 | United States | California | Shasta | 2/4/2021 | L. Newton |
| BME102242 | *Aptostichus barackobamai* | S Cow Creek Rd | 40.54 | -122.1131 | United States | California | Shasta | 2/24/2021 | L. Newton |
| BME102305 | *Aptostichus barackobamai* | CA-Hwy 128, S of Boonville | 38.8295 | -123.044 | United States | California | Sonoma | 3/15/2021 | L. Newton, E. Joachim |
| MY1098 | *Aptostichus barackobamai* | Hwy 3, near Douglas City | 40.64972 | -122.94109 | United States | California | Trinity | 03/15/2006 | M Hedin, J Starrett, S Thomas, R Keith, S Derkarabetian, M McCormack, D Marxsen |
| MY3025 | *Aptostichus barackobamai* | Orr Springs Rd, Ackerman Creek- 1st stream crossing W of Hwy 101 | 39.1807 | -123.23307 | United States | California | Mendocino | 03/14/2005 | J. Bond, A. Stockman, D. Beamer |
| MY3026 | *Aptostichus barackobamai* | Orr Springs Rd, 2.4 mi W of bridge | 39.19242 | -123.26595 | United States | California | Mendocino | 03/14/2005 | J. Bond, A. Stockman, D. Beamer |
| MY3027 | *Aptostichus barackobamai* | Orr Springs Rd, W of Hwy 101, 8.3 mi W of 1st bridge | 39.22932 | -123.34265 | United States | California | Mendocino | 03/14/2005 | J. Bond, A. Stockman, D. Beamer |
| MY3038 | *Aptostichus barackobamai* | Orr Springs Rd, 24.1 mi W of Ackerman Creek | 39.26095 | -123.5485 | United States | California | Mendocino | 03/14/2005 | J. Bond, A. Stockman, D. Beamer |
| MY3158 | *Aptostichus barackobamai* | County Rd 201, 4.2 mi N of JCT w/ Hwy 175 (201 is turnoff for Hopland Field STN, but do not turn onto University Dr.) | 39.02832 | -123.13034 | United States | California | Mendocino | 05/18/2005 | A.K. Stockman |
| MY3173 | *Aptostichus barackobamai* | Platina Rd, 2.9 mi NE of Hwy 36 | 40.36566 | -122.858 | United States | California | Shasta | 05/22/2005 | A. Stockman |
| MY3175 | *Aptostichus barackobamai* | Lower Springs Rd, 0.5 mi S of Hwy 299 | 40.58076 | -122.45019 | United States | California | Shasta | 05/23/2005 | A. Stockman |
| MY3621 | *Aptostichus barackobamai* | Hwy32, S of Deer Creek, 3.3mi SW Potato Patch CG | 40.45912 | -121.78287 | United States | California | Tehama | 12/19/2007 | M Hedin, J Starrett, D Leavitt |
| MY3622 | *Aptostichus barackobamai* | Hwy32, S of Deer Creek, 3.3mi SW Potato Patch CG | 40.15993 | -121.5704 | United States | California | Tehama | 12/19/2007 | M Hedin, J Starrett, D Leavitt |
| MY3803 | *Aptostichus barackobamai* | Cottonwood | 40.316774 | -122.34998 | United States | California | Tehama | 10/15/2009 | C.S. Will |
| MY729 | *Aptostichus barackobamai* | Sutter Buttes, Dean Place | 39.223066 | -121.78128 | United States | California | Sutter | 04/04/2003 | Hedin, Paquin, Starrett |
| BME102752 | *Aptostichus icenoglei* (Central) | Cleveland Forest Rd, Cleveland NF | 33.5289 | -117.3885 | United States | California | Riverside | 5/13/2021 | L. Newton, J. Starrett |
| BME102753 | *Aptostichus icenoglei* (Central) | Cleveland Forest Rd, Cleveland NF | 33.5289 | -117.3885 | United States | California | Riverside | 5/13/2021 | L. Newton, J. Starrett |
| MY2465 | *Aptostichus icenoglei* (Central) | North of Fallbrook on DeLuz Road | 33.41095 | -117.28984 | United States | California | San Diego | 01/30/2004 | J. Bond & M.Hedin |
| MY2467 | *Aptostichus icenoglei* (Central) | North of Fallbrook on DeLuz Road | 33.41095 | -117.28984 | United States | California | San Diego | 01/30/2004 | J. Bond & M.Hedin |
| MY2480 | *Aptostichus icenoglei* (Central) | Bautista Canyon, along Bautista Canyon Road | 33.70998 | -116.87756 | United States | California | Riverside | 02/01/2004 | J. Bond |
| MY2492 | *Aptostichus icenoglei* (Central) | Ortega HWY H74, ~1.7 miles North Orange Co/Riverside Co line | 33.61276 | -117.43462 | United States | California | Riverside | 02/02/2004 | J. Bond |
| MY2505 | *Aptostichus icenoglei* (Central) | Winchester, just east of Icenogle residence, end of Grand Ave | 33.71568 | -117.09365 | United States | California | Riverside | 01/29/2004 | J. Bond |
| MY2512 | *Aptostichus icenoglei* (Central) | Winchester, just east of Icenogle residence, end of Grand Ave | 33.71568 | -117.09365 | United States | California | Riverside | 01/29/2004 | J. Bond |
| MY2523 | *Aptostichus icenoglei* (Central) | Winchester, just east of Icenogle residence, end of Grand Ave | 33.71568 | -117.09365 | United States | California | Riverside | 01/29/2004 | J. Bond |
| MY2597 | *Aptostichus icenoglei* (Central) | Winchester, Leona Rd ~1.0 m South of intersection with Patton Ave | 33.67712 | -117.11578 | United States | California | Riverside | 03/13/2004 | J. Bond, W. Icenogle, et al |
| MY2668 | *Aptostichus icenoglei* (Central) | Cleveland National Forest, along H74 | 33.629719 | -117.42525 | United States | California | Riverside | 03/18/2004 | J. Bond, C. Spruill, D. Beamer |
| MY2669 | *Aptostichus icenoglei* (Central) | Cleveland National Forest, along H74 | 33.629719 | -117.42525 | United States | California | Riverside | 03/18/2004 | J. Bond, C. Spruill, D. Beamer |
| MY3776 | *Aptostichus icenoglei* (Central) | Winchester, Grand Ave ~.6 mile east intersection of Grand and Matthews | 33.714781 | -117.11009 | United States | California | Riverside | 05/17/2009 | J. Bond |
| MY3777 | *Aptostichus icenoglei* (Central) | Winchester, Grand Ave ~.6 mile east intersection of Grand and Matthews | 33.714781 | -117.11009 | United States | California | Riverside | 05/17/2009 | J. Bond |
| MY718 | *Aptostichus icenoglei* (Central) | De Luz Murrieta Road | 33.49565 | -117.24338 | United States | California | Riverside | 01/11/2003 | M. Hedin |
| BME102526 | *Aptostichus icenoglei* (North) | Angeles NF, Angeles Forest Hwy (N3) near jct w/ Big Tujunga Canyon Rd | 34.2907 | -118.1706 | United States | California | Los Angeles | 5/11/2021 | L. Newton, J. Bond, J. Starrett, L. Chamberland |
| BME102534 | *Aptostichus icenoglei* (North) | Cleghorn Rd, San Bernardino NF, Silverwood Lake Rec Area, managed by CA State Parks | 34.2853 | -117.3728 | United States | California | San Bernardino | 5/12/2021 | L. Newton, J. Bond, J. Starrett, L. Chamberland |
| BME102535 | *Aptostichus icenoglei* (North) | Cleghorn Rd, San Bernardino NF, Silverwood Lake Rec Area, managed by CA State Parks | 34.2853 | -117.3728 | United States | California | San Bernardino | 5/12/2021 | L. Newton, J. Bond, J. Starrett, L. Chamberland |
| BME102536 | *Aptostichus icenoglei* (North) | Cleghorn Rd, San Bernardino NF, Silverwood Lake Rec Area, managed by CA State Parks | 34.2853 | -117.3728 | United States | California | San Bernardino | 5/12/2021 | L. Newton, J. Bond, J. Starrett, L. Chamberland |
| BME102537 | *Aptostichus icenoglei* (North) | CA Hwy 138, San Bernardino NF | 34.2588 | -117.2944 | United States | California | San Bernardino | 5/12/2021 | L. Newton, J. Bond, J. Starrett, L. Chamberland |
| BME102748 | *Aptostichus icenoglei* (North) | CA Hwy 38 | 34.0767 | -117.0568 | United States | California | San Bernardino | 5/12/2021 | L. Newton, J. Bond, J. Starrett, L. Chamberland |
| MY2600 | *Aptostichus icenoglei* (North) | Puente Hills, intersection of Azusa & Tomich Rd | 33.98161 | -117.93351 | United States | California | Los Angeles | 03/14/2004 | J. Bond, C. Spruill, D. Beamer |
| MY3759 | *Aptostichus icenoglei* (North) | Mt. Baldy Rd, ~0.2 km N of jct w/ N Mountain Ave | 34.1773 | -117.6767 | United States | California | Los Angeles | 02/15/2009 | M Hedin, J Satler, J Starrett, C Richart |
| MY3763 | *Aptostichus icenoglei* (North) | Lytle Creek Rd, near Scotland | 34.244 | -117.4952 | United States | California | San Bernardino | 02/15/2009 | M Hedin, J Satler, J Starrett, C Richart |
| BME102828 | *Aptostichus icenoglei* (South) | CA Hwy 76, Morettis Junction | 33.2015 | -116.7118 | United States | California | San Diego | 6/22/2021 | L. Newton, J. Starrett, R. Ruedas, B. Gibson |
| BME102829 | *Aptostichus icenoglei* (South) | CA Hwy 76, Morettis Junction | 33.2015 | -116.7118 | United States | California | San Diego | 6/22/2021 | L. Newton, J. Starrett, R. Ruedas, B. Gibson |
| BME102830 | *Aptostichus icenoglei* (South) | CA Hwy 76, Morettis Junction | 33.2015 | -116.7118 | United States | California | San Diego | 6/22/2021 | L. Newton, J. Starrett, R. Ruedas, B. Gibson |
| BME102831 | *Aptostichus icenoglei* (South) | CA Hwy 76, Morettis Junction | 33.2015 | -116.7118 | United States | California | San Diego | 6/22/2021 | L. Newton, J. Starrett, R. Ruedas, B. Gibson |
| BME102832 | *Aptostichus icenoglei* (South) | CA Hwy 76, Morettis Junction | 33.2015 | -116.7118 | United States | California | San Diego | 6/22/2021 | L. Newton, J. Starrett, R. Ruedas, B. Gibson |
| BME102833 | *Aptostichus icenoglei* (South) | CA Hwy 76, Morettis Junction | 33.2015 | -116.7118 | United States | California | San Diego | 6/22/2021 | L. Newton, J. Starrett, R. Ruedas, B. Gibson |
| BME102837 | *Aptostichus icenoglei* (South) | CA Hwy 76, Cleveland NF, across from picnic area | 33.2533 | -116.7922 | United States | California | San Diego | 6/22/2021 | L. Newton, J. Starrett, R. Ruedas, B. Gibson |
| BME102842 | *Aptostichus icenoglei* (South) | Lyons Valley Rd | 32.7526 | -116.6715 | United States | California | San Diego | 6/23/2021 | L. Newton, J. Starrett, R. Ruedas |
| BME102844 | *Aptostichus icenoglei* (South) | Lyons Valley Rd | 32.7526 | -116.6715 | United States | California | San Diego | 6/23/2021 | L. Newton, J. Starrett, R. Ruedas |
| BME102845 | *Aptostichus icenoglei* (South) | Otay Mtn Ecological Reserve, ~1 mile up trail | 32.6365 | -116.884 | United States | California | San Diego | 6/23/2021 | L. Newton, J. Starrett, R. Ruedas, M. Hedin |
| BME102847 | *Aptostichus icenoglei* (South) | El Monte Rd | 32.8838 | -116.8214 | United States | California | San Diego | 6/24/2021 | L. Newton, J. Starrett, M. Hedin |
| BME102851 | *Aptostichus icenoglei* (South) | Torrey Pines State Reserve Extension, Mar Scenic Trail | 32.9459 | -117.2543 | United States | California | San Diego | 6/24/2021 | L. Newton, J. Starrett |
| MY305 | *Aptostichus icenoglei* (South) | El Monte Park Rd. | 32.88395 | -116.82145 | United States | California | San Diego | 01/19/2002 | M.C. Hedin |
| MY306 | *Aptostichus icenoglei* (South) | El Monte Park Rd. | 32.88395 | -116.82145 | United States | California | San Diego | 01/19/2002 | M.C. Hedin |
| MY3635 | *Aptostichus icenoglei* (South) | E. Lakeside between El Monte Park & entrance to El Capitan Res, El Monte Rd | 32.88369 | -116.82239 | United States | California | San Diego | 02/23/2008 | M.C. Hedin |
| MY719 | *Aptostichus icenoglei* (South) | Nate Harrison Grade Road, 2.1 mi E jnct w/HWY 76 | 33.32697 | -116.96523 | United States | California | San Diego | 01/11/2003 | M. Hedin |
| MY3824 | *Aptostichus isabella* | Erskine Creek Rd., 3.5 mi. E of int. w/ Lake Isabella Blvd., E of Bodfish | 35.5689 | -118.4383 | United States | California | Kern | 10/08/2010 | J. Satler |

Supplemental Table 2. Morphological measurements of each *A. icenoglei* lineage.

| Voucher ID | Clade/Group | Carapace L | Carapace W | Posterior eye row W | Sternum L | Sternum W | Labium L | Labium W | Femur I | Patella I | Tibia I | Metatarsus I | Tarsus I | Femur IV | Patella IV | Tibia IV | Metatarsus IV | Tarsus IV | Tibia I spines prolateral | Tibia I spines retrolateral | Tibia I spines retrolateral distal | Patella I spines prolateral | Metatarsus I spines prolateral | Metatarsus I spines retrolateral | Palpal tibia L | Palpal tibia W | Bulb L |
| --- | --- | --- | --- | --- | --- | --- | --- | --- | --- | --- | --- | --- | --- | --- | --- | --- | --- | --- | --- | --- | --- | --- | --- | --- | --- | --- | --- |
| AP 886 | South | 4.784 | 3.925 | 0.863 | 2.657 | 2.213 | 0.355 | 0.661 | 4.793 | 2.405 | 3.372 | 3.12 | 2.114 | 4.811 | 2.072 | 4.2 | 4.668 | 2.588 | 3 | 2 | 6 | 1 | 2 | 0 | 1.873 | 0.727 | 1.005 |
| AP 912b | South | 5.811 | 4.749 | 1.026 | 3.167 | 2.493 | 0.417 | 0.834 | 5.393 | 2.663 | 3.946 | 3.707 | 2.843 | 5.619 | 2.525 | 4.814 | 5.288 | 3.106 | 3 | 2 | 7 | 1 | 2 | 0 | 2.225 | 0.764 | 1.057 |
| AP 912a | South | 4.929 | 3.925 | 0.874 | 2.816 | 2.241 | 0.373 | 0.621 | 4.825 | 2.308 | 3.556 | 3.277 | 2.49 | 5.154 | 2.196 | 4.535 | 4.613 | 2.731 | 3 | 2 | 8 | 1 | 2 | 0 | 2.041 | 0.706 | 1.025 |
| AP 1092 | South | 5.603 | 4.69 | 1.009 | 3.142 | 2.567 | 0.358 | 0.795 | 5.363 | 2.627 | 3.968 | 3.809 | 2.796 | 5.788 | 2.425 | 4.729 | 5.137 | 2.602 | 2 | 0 | 8 | 1 | 1 | 0 | 2.306 | 0.794 | 1.163 |
| AP 1069 | South | 5.836 | 4.741 | 0.977 | 3.26 | 2.55 | 0.443 | 0.755 | 5.812 | 2.905 | 4.141 | 4.067 | 2.961 | 5.658 | 2.603 | 5.129 | 5.619 | 2.78 | 3 | 2 | 7 | 1 | 1 | 0 | 2.492 | 0.786 | 1.168 |
| AP 941 | South | 5.197 | 4.298 | 0.853 | 2.859 | 2.229 | 0.362 | 0.568 | 4.948 | 2.442 | 3.429 | 3.279 | 2.463 | 5.195 | 2.35 | 4.499 | 4.769 | 2.397 | 3 | 2 | 7 | 1 | 1 | 0 | 2.031 | 0.766 | 1.038 |
| AP 019 | South | 4.947 | 3.932 | 0.888 | 2.633 | 2.196 | 0.33 | 0.679 | 4.675 | 2.23 | 3.378 | 3.335 | 2.339 | 4.88 | 2.045 | 4.204 | 4.627 | 2.329 | 3 | 2 | 7 | 1 | 2 | 0 | 1.995 | 0.686 | 0.884 |
| AP 914 | South | 4.801 | 3.911 | 0.831 | 2.622 | 2.106 | 0.351 | 0.648 | 4.693 | 2.245 | 3.248 | 3.261 | 2.346 | 4.852 | 2.07 | 4.249 | 4.484 | 2.523 | 2 | 3 | 6 | 0 | 0 | 1 | 1.974 | 0.678 | 0.858 |
| AP 878 | South | 5.868 | 4.526 | 0.901 | 3.01 | 2.367 | 0.481 | 0.78 | 5.146 | 2.656 | 3.682 | 3.526 | 2.857 | 5.468 | 2.533 | 4.406 | 4.839 | 2.733 | 3 | 2 | 5 | 1 | 2 | 0 | 2.16 | 0.765 | 1.086 |
| AP 927 | South | 4.577 | 3.84 | 0.812 | 2.51 | 2.05 | 0.413 | 0.641 | 4.258 | 2.123 | 3.224 | 3.234 | 2.263 | 4.562 | 2.027 | 3.981 | 4.421 | 2.369 | 2 | 0 | 8 | 0 | 1 | 0 | 1.894 | 0.679 | 0.876 |
| AP 989 | Central | 4.717 | 3.883 | 0.883 | 2.745 | 1.988 | 0.338 | 0.717 | 5.095 | 2.413 | 3.893 | 3.655 | 2.594 | 5.136 | 2.24 | 4.496 | 5.131 | 2.596 | 3 | 2 | 7 | 1 | 1 | 0 | 2.137 | 0.682 | 1.024 |
| AP 825 | Central | 5.33 | 4.35 | 0.88 | 3.028 | 2.272 | 0.391 | 0.723 | 5.231 | 2.655 | 3.992 | 3.767 | 2.683 | 5.316 | 2.302 | 4.491 | 5.068 | 2.647 | 3 | 2 | 7 | 1 | 2 | 0 | 2.129 | 0.727 | 0.802 |
| AP 1231 | Central | 4.778 | 3.918 | 0.85 | 2.874 | 2.233 | 0.272 | 0.72 | 4.978 | 2.409 | 3.696 | 3.48 | 2.616 | 5.241 | 2.108 | 4.478 | 5.053 | 2.782 | 2 | 2 | 7 | 0 | 1 | 0 | 2.073 | 0.662 | 0.945 |
| AP 1113 | Central | 5.983 | 4.866 | 1.007 | 3.14 | 2.39 | 0.341 | 0.754 | 5.448 | 2.747 | 4.142 | 3.589 | 2.586 | 5.678 | 2.377 | 4.871 | 5.415 | 3.015 | 3 | 2 | 7 | 1 | 1 | 0 | 2.224 | 0.762 | 1.07 |
| AP 979 | Central | 5.88 | 4.715 | 1.032 | 3.257 | 2.608 | 0.408 | 0.753 | 5.712 | 2.883 | 4.507 | 4.163 | 2.969 | 5.605 | 2.557 | 4.776 | 5.739 | 3.024 | 3 | 2 | 9 | 2 | 2 | 0 | 2.35 | 0.749 | 1.102 |
| AP 963 | Central | 5.768 | 4.621 | 0.97 | 3.224 | 2.405 | 0.381 | 0.757 | 5.591 | 2.695 | 4.168 | 4.114 | 2.82 | 5.667 | 2.375 | 4.664 | 5.616 | 2.694 | 2 | 2 | 7 | 1 | 3 | 0 | 2.205 | 0.741 | 1.087 |
| AP 1134 | Central | 4.541 | 3.811 | 0.772 | 2.627 | 2.08 | 0.232 | 0.633 | 4.68 | 2.292 | 3.604 | 3.474 | 2.535 | 4.752 | 1.876 | 4.141 | 4.56 | 2.538 | 3 | 2 | 7 | 0 | 1 | 0 | 1.976 | 0.645 | 0.912 |
| AP 875 | Central | 4.846 | 3.787 | 0.854 | 2.71 | 2.006 | 0.298 | 0.553 | 4.657 | 2.222 | 3.61 | 3.556 | 2.261 | 4.741 | 2.052 | 4.267 | 4.562 | 2.557 | 3 | 2 | 8 | 0 | 1 | 0 | 2.037 | 0.649 | 0.951 |
| AP 1144 | Central | 5.88 | 4.935 | 1.047 | 3.359 | 2.516 | 0.303 | 0.783 | 5.543 | 2.743 | 4.298 | 4.171 | 2.813 | 5.764 | 2.454 | 4.99 | 5.46 | 3.035 | 2 | 2 | 8 | 2 | 2 | 0 | 2.335 | 0.779 | 1.079 |
| AP 899 | Central | 5.323 | 4.46 | 0.905 | 3.117 | 2.342 | 0.386 | 0.707 | 5.29 | 2.684 | 4.085 | 3.916 | 2.759 | 5.532 | 2.403 | 4.774 | 5.091 | 2.604 | 2 | 1 | 8 | 0 | 1 | 0 | 2.275 | 0.743 | 1.067 |
| AP 1215 | North | 6.029 | 4.809 | 0.964 | 3.273 | 2.561 | 0.399 | 0.787 | 5.479 | 2.504 | 4.203 | 4.07 | 2.787 | 6.028 | 2.652 | 5.078 | 5.757 | 2.994 | 1 | 1 | 9 | 1 | 0 | 0 | 2.366 | 0.771 | 1.173 |
| AP 1171 | North | 5.016 | 4.081 | 0.856 | 2.917 | 2.141 | 0.327 | 0.851 | 4.99 | 2.38 | 3.993 | 3.479 | 2.57 | 4.626 | 2.059 | 4.502 | 5.227 | 2.684 | 2 | 2 | 6 | 0 | 1 | 0 | 2.143 | 0.687 | 0.967 |
| AP 32 | North | 5.43 | 4.303 | 0.933 | 2.888 | 2.334 | 0.321 | 0.762 | 5.379 | 2.65 | 3.968 | 3.899 | 2.617 | 5.484 | 2.323 | 4.715 | 5.245 | 3.036 | 2 | 2 | 8 | 1 | 1 | 0 | 2.216 | 0.682 | 1.035 |
| AP 1173 | North | 4.951 | 4.205 | 0.956 | 2.984 | 2.107 | 0.394 | 0.731 | 5.309 | 2.41 | 4.036 | 3.769 | 2.669 | 5.462 | 2.274 | 4.609 | 5.378 | 2.906 | 2 | 0 | 6 | 0 | 1 | 0 | 2.287 | 0.726 | 1.059 |
| AP 49 | North | 5.935 | 4.794 | 0.92 | 3.37 | 2.367 | 0.353 | 0.68 | 5.651 | 2.717 | 4.37 | 3.925 | 2.844 | 5.622 | 2.441 | 5.111 | 5.732 | 2.902 | 3 | 2 | 8 | 1 | 1 | 0 | 2.351 | 0.776 | 0.992 |
| AP 021b | North | 4.157 | 3.492 | 0.812 | 2.468 | 1.783 | 0.335 | 0.576 | 4.279 | 2.252 | 3.439 | 3.207 | 2.238 | 4.142 | 1.878 | 3.805 | 4.277 | 2.386 | 2 | 0 | 6 | 0 | 2 | 0 | 1.792 | 0.613 | 0.848 |
| AP 1242 | North | 5.275 | 4.34 | 0.965 | 3.006 | 1.791 | 0.245 | 0.734 | 5.254 | 2.513 | 4.003 | 3.743 | 2.735 | 5.489 | 2.377 | 4.696 | 5.217 | 2.642 | 2 | 1 | 8 | 1 | 1 | 0 | 1.969 | 0.718 | 0.893 |
| AP 1168 | North | 5.361 | 4.294 | 0.929 | 2.957 | 2.258 | 0.373 | 0.708 | 4.841 | 2.53 | 3.688 | 3.424 | 2.487 | 5.019 | 2.038 | 4.351 | 4.799 | 2.326 | 2 | 2 | 8 | 0 | 1 | 0 | 2.041 | 0.672 | 1.022 |
| AP 25 | North | 6.082 | 4.768 | 0.986 | 3.277 | 2.622 | 0.403 | 0.847 | 5.809 | 2.32 | 4.642 | 4.051 | 2.983 | 5.9 | 2.115 | 5.109 | 5.758 | 3.052 | 3 | 3 | 6 | 2 | 1 | 0 | 2.345 | 0.727 | 1.124 |
| AP 21a | North | 5.744 | 4.614 | 0.968 | 3.165 | 2.532 | 0.368 | 0.813 | 5.017 | 2.596 | 3.688 | 3.685 | 2.549 | 5.418 | 2.448 | 4.547 | 4.784 | 2.79 | 3 | 1 | 7 | 1 | 1 | 0 | 2.128 | 0.745 | 1.073 |

Supplemental Table 3. Bioclimatic variables used and their percent contribution to each species distribution model.

| Bioclimatic Variables | % Contribution |  |  |  |
| --- | --- | --- | --- | --- |
|  | **North** | **Central+South** | **Central** | **South** |
| bio2 - mean diurnal range (mean of monthly (max temp - min temp)) | 16.6163 | 0.8843 | 2.2109 | 0 |
| bio4 - temperature seasonality (standard deviation * 100) | 0 | 25.2361 | 15.3477 | 45.2929 |
| bio8 - mean temperature of wettest quarter | 0 | 1.7744 | 0.2544 | 0.0687 |
| bio9 - mean temperature of driest quarter | 6.4207 | 0.8392 | 0.3835 | 0.1458 |
| bio11 - mean temperature of coldest quarter | 0 | 13.4691 | 13.5075 | 18.3141 |
| bio14 - precipitation of driest month | 2.5498 | 24.776 | 50.2496 | 23.4304 |
| bio15 - precipitation seasonality (coefficient of variation) | 16.6163 | 13.2709 | 3.4537 | 4.2049 |
| bio17 - precipitation of driest quarter | 0.2437 | 0.2556 | 0 | 0.2486 |
| bio18 - precipitation of warmest quarter | 0 | 4.1435 | 2.0492 | 7.0691 |
| bio19 - precipitation of coldest quarter | 57.5531 | 15.3509 | 12.5434 | 1.2254 |

Supplemental Table 4. MaxEnt model parameters and stats for the North lineage.

| tune.args | auc.train | auc.diff.avg | auc.diff.sd | auc.val.avg | auc.val.sd | AICc | delta.AICc |
| --- | --- | --- | --- | --- | --- | --- | --- |
| rm.1_fc.L | 0.96298519 | 0.04779466 | 0.39232578 | 0.94986481 | 0.44556522 | 549.884713 | 12.0839262 |
| rm.1.5_fc.L | 0.96284444 | 0.04776766 | 0.41209155 | 0.94954444 | 0.46221189 | 551.227616 | 13.4268291 |
| rm.2_fc.L | 0.96262963 | 0.04778177 | 0.43105457 | 0.94897407 | 0.47849848 | 548.42725 | 10.6264626 |
| rm.2.5_fc.L | 0.96271481 | 0.04741702 | 0.44144802 | 0.94846296 | 0.48655638 | 546.156036 | 8.35524882 |
| rm.3_fc.L | 0.96268889 | 0.04739466 | 0.4543244 | 0.94772963 | 0.49741888 | 547.363022 | 9.56223518 |
| rm.1_fc.LQ | 0.96781111 | 0.05975114 | 0.68285435 | 0.94010741 | 0.72417914 | 554.746197 | 16.9454102 |
| rm.1.5_fc.LQ | 0.96528148 | 0.05440541 | 0.54778806 | 0.94315926 | 0.59321122 | 550.943034 | 13.1422466 |
| rm.2_fc.LQ | 0.96424259 | 0.05245328 | 0.52863286 | 0.94455 | 0.57286457 | 550.280265 | 12.4794776 |
| rm.2.5_fc.LQ | 0.96412407 | 0.05062265 | 0.51013538 | 0.94558704 | 0.55301514 | 551.468502 | 13.6677154 |
| rm.3_fc.LQ | 0.96406296 | 0.04926873 | 0.49548591 | 0.94597222 | 0.53704121 | 549.024674 | 11.2238872 |
| rm.1_fc.H | 0.97625556 | 0.03319651 | 0.26049492 | 0.96385556 | 0.2967624 | 800.702884 | 262.902097 |
| rm.1.5_fc.H | 0.97480556 | 0.03364316 | 0.23891821 | 0.96552037 | 0.28286583 | 610.709568 | 72.9087807 |
| rm.2_fc.H | 0.97346667 | 0.03504088 | 0.23858597 | 0.96484444 | 0.28664047 | 599.250919 | 61.4501316 |
| rm.2.5_fc.H | 0.97212963 | 0.03621047 | 0.2425729 | 0.96404074 | 0.29300541 | 619.379255 | 81.5784683 |
| rm.3_fc.H | 0.97111481 | 0.03647678 | 0.23972866 | 0.96426667 | 0.29178159 | 575.82525 | 38.0244628 |
| rm.1_fc.LQH | 0.97627778 | 0.06068362 | 0.88744138 | 0.93574815 | 0.91134538 | 625.579344 | 87.7785568 |
| rm.1.5_fc.LQH | 0.97352222 | 0.0609995 | 0.8428971 | 0.93736667 | 0.87159091 | 544.287204 | 6.48641697 |
| rm.2_fc.LQH | 0.97290741 | 0.05935178 | 0.78053554 | 0.94001852 | 0.81204706 | 546.738124 | 8.93733695 |
| rm.2.5_fc.LQH | 0.97217037 | 0.05894117 | 0.75609569 | 0.94045556 | 0.78912233 | 549.277034 | 11.4762472 |
| rm.3_fc.LQH | 0.97152963 | 0.05803155 | 0.71873611 | 0.9410963 | 0.75327525 | 551.987819 | 14.1870318 |
| rm.1_fc.LQHP | 0.97868148 | 0.04548362 | 0.54655157 | 0.94965926 | 0.57090023 | 589.070752 | 51.2699646 |
| rm.1.5_fc.LQHP | 0.97721852 | 0.04245271 | 0.45115177 | 0.95376667 | 0.48092442 | 555.463005 | 17.6622184 |
| rm.2_fc.LQHP | 0.97647407 | 0.04082892 | 0.38955964 | 0.95597037 | 0.42350678 | 542.574317 | 4.77352976 |
| rm.2.5_fc.LQHP | 0.97525926 | 0.04002756 | 0.34951821 | 0.95741667 | 0.38787051 | 546.652985 | 8.85219787 |
| rm.3_fc.LQHP | 0.97374815 | 0.03883967 | 0.31722382 | 0.95823889 | 0.35792122 | 537.800787 | 0 |
| rm.1_fc.LQHPT | 0.98247593 | 0.04396959 | 0.52522461 | 0.95121296 | 0.54702761 | 592.628259 | 54.8274718 |
| rm.1.5_fc.LQHPT | 0.97866111 | 0.04078718 | 0.43786459 | 0.95493519 | 0.46567261 | 557.37636 | 19.5755732 |
| rm.2_fc.LQHPT | 0.97709815 | 0.03885855 | 0.38079042 | 0.9576537 | 0.41266484 | 538.632034 | 0.83124731 |
| rm.2.5_fc.LQHPT | 0.97601852 | 0.03814338 | 0.34448916 | 0.95852778 | 0.37962652 | 543.624951 | 5.82416433 |
| rm.3_fc.LQHPT | 0.97480741 | 0.03745919 | 0.3164356 | 0.95910556 | 0.35428705 | 543.029111 | 5.22832447 |

Supplemental Table 5. MaxEnt model parameters and stats for the Central+South lineage.

| tune.args | auc.train | auc.diff.avg | auc.diff.sd | auc.val.avg | auc.val.sd | AICc | delta.AICc |
| --- | --- | --- | --- | --- | --- | --- | --- |
| rm.1_fc.L | 0.92911282 | 0.05412564 | 0.37713394 | 0.92583974 | 0.59981181 | 1646.16976 | 59.4997409 |
| rm.1.5_fc.L | 0.92856154 | 0.05505778 | 0.38588845 | 0.92527179 | 0.61179284 | 1647.51683 | 60.8468182 |
| rm.2_fc.L | 0.92798974 | 0.05613982 | 0.39901875 | 0.92442692 | 0.62719083 | 1651.56762 | 64.8976065 |
| rm.2.5_fc.L | 0.92736154 | 0.05729902 | 0.4141694 | 0.92357692 | 0.64526349 | 1653.33674 | 66.6667211 |
| rm.3_fc.L | 0.92640769 | 0.05833722 | 0.42296837 | 0.92314103 | 0.65746121 | 1655.22383 | 68.5538101 |
| rm.1_fc.LQ | 0.94863462 | 0.03683689 | 0.25410464 | 0.94496026 | 0.405763 | 1607.05714 | 20.3871202 |
| rm.1.5_fc.LQ | 0.94661538 | 0.04022816 | 0.27096206 | 0.94217308 | 0.43982405 | 1613.0727 | 26.4026876 |
| rm.2_fc.LQ | 0.94381667 | 0.04285914 | 0.27828473 | 0.93974359 | 0.46202823 | 1620.86462 | 34.1946018 |
| rm.2.5_fc.LQ | 0.94223718 | 0.04419367 | 0.2866835 | 0.93823974 | 0.47628745 | 1622.66543 | 35.9954114 |
| rm.3_fc.LQ | 0.940775 | 0.04562885 | 0.29879324 | 0.93702051 | 0.4939026 | 1623.79741 | 37.1273963 |
| rm.1_fc.H | 0.96558654 | 0.03626742 | 0.23560758 | 0.95934359 | 0.38959281 | 1586.67002 | 0 |
| rm.1.5_fc.H | 0.96197628 | 0.03895958 | 0.24678472 | 0.95607179 | 0.41530767 | 1594.94041 | 8.27039099 |
| rm.2_fc.H | 0.95915577 | 0.04128498 | 0.2523317 | 0.95430769 | 0.43584386 | 1603.35359 | 16.6835754 |
| rm.2.5_fc.H | 0.95743782 | 0.04273198 | 0.26675213 | 0.95216731 | 0.45452108 | 1616.19434 | 29.5243203 |
| rm.3_fc.H | 0.95523974 | 0.0437811 | 0.27642794 | 0.95107756 | 0.46840257 | 1615.53699 | 28.8669743 |
| rm.1_fc.LQH | 0.96825 | 0.03306742 | 0.23550624 | 0.96031154 | 0.36483608 | 1610.04176 | 23.3717456 |
| rm.1.5_fc.LQH | 0.96045256 | 0.0363532 | 0.22514301 | 0.95490769 | 0.38406344 | 1599.51689 | 12.8468697 |
| rm.2_fc.LQH | 0.95580641 | 0.03885006 | 0.23790617 | 0.95087692 | 0.40990389 | 1599.93957 | 13.2695547 |
| rm.2.5_fc.LQH | 0.95275641 | 0.0405723 | 0.24981235 | 0.94789103 | 0.42889921 | 1610.71045 | 24.0404373 |
| rm.3_fc.LQH | 0.9494141 | 0.04133966 | 0.26177159 | 0.94503077 | 0.44186619 | 1610.90905 | 24.2390338 |
| rm.1_fc.LQHP | 0.96937692 | 0.03249883 | 0.25166605 | 0.96298333 | 0.37300608 | 1615.34787 | 28.6778595 |
| rm.1.5_fc.LQHP | 0.96518462 | 0.03663112 | 0.2479068 | 0.95817564 | 0.39929461 | 1603.77793 | 17.1079143 |
| rm.2_fc.LQHP | 0.95884615 | 0.04104854 | 0.24639402 | 0.95258718 | 0.43096947 | 1602.94789 | 16.2778786 |
| rm.2.5_fc.LQHP | 0.95352885 | 0.04275626 | 0.25045322 | 0.94949744 | 0.44499983 | 1601.76718 | 15.0971681 |
| rm.3_fc.LQHP | 0.95168654 | 0.04369592 | 0.25861651 | 0.94778077 | 0.45797318 | 1613.28568 | 26.6156599 |
| rm.1_fc.LQHPT | 0.97612308 | 0.03064732 | 0.26863002 | 0.96570256 | 0.36609556 | 1640.71733 | 54.0473164 |
| rm.1.5_fc.LQHPT | 0.97025641 | 0.03661325 | 0.26295824 | 0.96038077 | 0.40466573 | 1604.15539 | 17.4853777 |
| rm.2_fc.LQHPT | 0.96234359 | 0.04159524 | 0.26329192 | 0.95433718 | 0.44237814 | 1608.32087 | 21.6508516 |
| rm.2.5_fc.LQHPT | 0.95607692 | 0.04372766 | 0.26134937 | 0.95079744 | 0.45706018 | 1611.8483 | 25.1782834 |
| rm.3_fc.LQHPT | 0.95344487 | 0.04442069 | 0.26345806 | 0.94869936 | 0.46493385 | 1608.30791 | 21.6378941 |

Supplemental Table 6. MaxEnt model parameters and stats for the Central lineage.

| tune.args | auc.train | auc.diff.avg | auc.diff.sd | auc.val.avg | auc.val.sd | AICc | delta.AICc |
| --- | --- | --- | --- | --- | --- | --- | --- |
| rm.1_fc.L | 0.94392917 | 0.05436444 | 0.38823508 | 0.93707639 | 0.49559136 | 756.962129 | 43.7023226 |
| rm.1.5_fc.L | 0.94371528 | 0.05254405 | 0.35560723 | 0.93831528 | 0.46474413 | 751.777257 | 38.5174515 |
| rm.2_fc.L | 0.9444625 | 0.05111048 | 0.33181239 | 0.93947361 | 0.44160104 | 746.196125 | 32.9363186 |
| rm.2.5_fc.L | 0.9451375 | 0.04993488 | 0.31451865 | 0.94029583 | 0.42411142 | 746.6486 | 33.3887939 |
| rm.3_fc.L | 0.94570417 | 0.04899579 | 0.29550739 | 0.94121111 | 0.40613788 | 747.155691 | 33.895885 |
| rm.1_fc.LQ | 0.95884583 | 0.04810944 | 0.38595911 | 0.94790694 | 0.4645682 | 735.644985 | 22.3851792 |
| rm.1.5_fc.LQ | 0.95670139 | 0.04575119 | 0.26779885 | 0.94944028 | 0.37004863 | 733.637027 | 20.3772213 |
| rm.2_fc.LQ | 0.95464306 | 0.04521024 | 0.21084075 | 0.94907917 | 0.33196685 | 738.301603 | 25.0417974 |
| rm.2.5_fc.LQ | 0.95282361 | 0.04583095 | 0.20294592 | 0.94775417 | 0.32964267 | 738.643526 | 25.3837197 |
| rm.3_fc.LQ | 0.95170694 | 0.04594579 | 0.19698505 | 0.94688472 | 0.32739011 | 741.623794 | 28.3639885 |
| rm.1_fc.H | 0.97693056 | 0.03032964 | 0.29891764 | 0.96733611 | 0.33858933 | 734.366018 | 21.1062119 |
| rm.1.5_fc.H | 0.97440972 | 0.02938131 | 0.22805616 | 0.96825694 | 0.27845304 | 731.189471 | 17.9296647 |
| rm.2_fc.H | 0.97243333 | 0.02899516 | 0.17353873 | 0.96816528 | 0.23652478 | 748.013238 | 34.7534322 |
| rm.2.5_fc.H | 0.97085972 | 0.02955675 | 0.14845985 | 0.96739444 | 0.22265643 | 730.539078 | 17.2792717 |
| rm.3_fc.H | 0.96905417 | 0.0306956 | 0.13115688 | 0.96583194 | 0.21758223 | 720.77335 | 7.51354366 |
| rm.1_fc.LQH | 0.97620972 | 0.03230952 | 0.40324616 | 0.96520972 | 0.43563893 | 718.511062 | 5.25125584 |
| rm.1.5_fc.LQH | 0.97142639 | 0.03413754 | 0.34938033 | 0.96354306 | 0.39415834 | 726.325952 | 13.0661463 |
| rm.2_fc.LQH | 0.96932639 | 0.0347119 | 0.31015599 | 0.96294028 | 0.36246905 | 713.259806 | 0 |
| rm.2.5_fc.LQH | 0.96773056 | 0.0354073 | 0.27681684 | 0.96195417 | 0.33739251 | 718.845123 | 5.58531706 |
| rm.3_fc.LQH | 0.96600694 | 0.03683694 | 0.24356585 | 0.96018472 | 0.31694246 | 724.534392 | 11.274586 |
| rm.1_fc.LQHP | 0.97774306 | 0.03209556 | 0.4207485 | 0.96619306 | 0.44983496 | 715.39198 | 2.13217427 |
| rm.1.5_fc.LQHP | 0.97397361 | 0.03300151 | 0.34557872 | 0.96504583 | 0.38662962 | 723.331666 | 10.0718604 |
| rm.2_fc.LQHP | 0.97037083 | 0.03299587 | 0.24351572 | 0.96475139 | 0.30373822 | 719.036143 | 5.77633695 |
| rm.2.5_fc.LQHP | 0.96798472 | 0.03501774 | 0.19104318 | 0.96200417 | 0.27374608 | 723.046475 | 9.7866694 |
| rm.3_fc.LQHP | 0.96390139 | 0.03802901 | 0.16164599 | 0.95913611 | 0.26677632 | 726.772875 | 13.5130692 |
| rm.1_fc.LQHPT | 0.98467361 | 0.02852258 | 0.36583201 | 0.9722625 | 0.39066346 | 910.53346 | 197.273654 |
| rm.1.5_fc.LQHPT | 0.9780625 | 0.03395155 | 0.35332005 | 0.9658625 | 0.39366389 | 760.193986 | 46.9341797 |
| rm.2_fc.LQHPT | 0.9717625 | 0.03402603 | 0.25615263 | 0.9649125 | 0.31464302 | 729.534736 | 16.2749305 |
| rm.2.5_fc.LQHPT | 0.96945972 | 0.03523845 | 0.20869755 | 0.96299861 | 0.28452331 | 728.29766 | 15.0378536 |
| rm.3_fc.LQHPT | 0.96573194 | 0.0381873 | 0.17214475 | 0.95976528 | 0.27177802 | 728.416082 | 15.1562756 |

Supplemental Table 7. MaxEnt model parameters and stats for the South lineage.

| tune.args | auc.train | auc.diff.avg | auc.diff.sd | auc.val.avg | auc.val.sd | AICc | delta.AICc |
| --- | --- | --- | --- | --- | --- | --- | --- |
| rm.1_fc.L | 0.94225833 | 0.05151934 | 0.25679548 | 0.93773452 | 0.40874048 | 876.996361 | 64.9941473 |
| rm.1.5_fc.L | 0.94006548 | 0.05304936 | 0.26698976 | 0.93599881 | 0.42268379 | 877.076306 | 65.0740923 |
| rm.2_fc.L | 0.93755595 | 0.05571545 | 0.28927862 | 0.9334869 | 0.44992052 | 880.865404 | 68.8631902 |
| rm.2.5_fc.L | 0.93456071 | 0.05840572 | 0.31384222 | 0.93119167 | 0.47778224 | 881.805619 | 69.8034049 |
| rm.3_fc.L | 0.9332369 | 0.06035261 | 0.33290551 | 0.92965595 | 0.50030345 | 880.767731 | 68.7655171 |
| rm.1_fc.LQ | 0.95866071 | 0.04852573 | 0.29015249 | 0.95230119 | 0.41654092 | 848.079681 | 36.0774667 |
| rm.1.5_fc.LQ | 0.95512976 | 0.05079559 | 0.28919573 | 0.94959167 | 0.42551871 | 856.39616 | 44.3939457 |
| rm.2_fc.LQ | 0.9534869 | 0.05102143 | 0.28404405 | 0.94854167 | 0.42391624 | 851.858213 | 39.8559988 |
| rm.2.5_fc.LQ | 0.95104167 | 0.05218275 | 0.28263564 | 0.9471869 | 0.42825585 | 857.959429 | 45.9572151 |
| rm.3_fc.LQ | 0.94833214 | 0.05454518 | 0.29192195 | 0.94467024 | 0.44502423 | 860.869967 | 48.8677533 |
| rm.1_fc.H | 0.98799048 | 0.01473844 | 0.10266451 | 0.98340952 | 0.13534538 | 839.214609 | 27.2123954 |
| rm.1.5_fc.H | 0.98280595 | 0.02193365 | 0.14505931 | 0.97715595 | 0.20174876 | 825.66722 | 13.6650059 |
| rm.2_fc.H | 0.97451548 | 0.03596672 | 0.22828974 | 0.96646548 | 0.32800609 | 834.411326 | 22.4091117 |
| rm.2.5_fc.H | 0.96581071 | 0.04600973 | 0.24923692 | 0.96179286 | 0.38400719 | 835.591702 | 23.5894875 |
| rm.3_fc.H | 0.96168214 | 0.05136713 | 0.26768145 | 0.95894048 | 0.41928127 | 832.123818 | 20.1216038 |
| rm.1_fc.LQH | 0.98632976 | 0.01798043 | 0.13089073 | 0.98057024 | 0.16970645 | 1042.71494 | 230.712729 |
| rm.1.5_fc.LQH | 0.98009405 | 0.02388508 | 0.14073166 | 0.97668214 | 0.20475392 | 819.446798 | 7.44458451 |
| rm.2_fc.LQH | 0.97445357 | 0.03320064 | 0.18800132 | 0.96964643 | 0.28026933 | 821.98997 | 9.9877559 |
| rm.2.5_fc.LQH | 0.96894405 | 0.04078162 | 0.22297546 | 0.96447381 | 0.33816488 | 826.066254 | 14.06404 |
| rm.3_fc.LQH | 0.96325833 | 0.04880215 | 0.26867206 | 0.95870833 | 0.40832745 | 825.616507 | 13.6142935 |
| rm.1_fc.LQHP | 0.98751429 | 0.01416434 | 0.1024044 | 0.98396905 | 0.13310384 | 870.109813 | 58.1075988 |
| rm.1.5_fc.LQHP | 0.98442976 | 0.01877021 | 0.11295378 | 0.97981548 | 0.16312547 | 837.989076 | 25.9868615 |
| rm.2_fc.LQHP | 0.97871786 | 0.02587189 | 0.14151122 | 0.975125 | 0.21636214 | 812.002214 | 0 |
| rm.2.5_fc.LQHP | 0.97289881 | 0.03396806 | 0.17556758 | 0.96996071 | 0.27542241 | 822.484896 | 10.4826825 |
| rm.3_fc.LQHP | 0.96955833 | 0.03746969 | 0.19035082 | 0.96752262 | 0.30098163 | 817.524504 | 5.52229034 |
| rm.1_fc.LQHPT | 0.98948095 | 0.01516626 | 0.11778149 | 0.98348095 | 0.14678495 | 813.672994 | 1.67077974 |
| rm.1.5_fc.LQHPT | 0.98634405 | 0.01822358 | 0.11884915 | 0.98079881 | 0.1622668 | 826.009618 | 14.0074041 |
| rm.2_fc.LQHPT | 0.98126429 | 0.02477468 | 0.13486162 | 0.97561548 | 0.20669472 | 830.452541 | 18.4503267 |
| rm.2.5_fc.LQHPT | 0.97460595 | 0.0320628 | 0.16397314 | 0.97108929 | 0.25980884 | 817.959958 | 5.95774382 |
| rm.3_fc.LQHPT | 0.97089167 | 0.03650499 | 0.18686754 | 0.96787738 | 0.29544553 | 821.706309 | 9.70409497 |

**Supplemental Figures**

Supplemental Figure 1. 50p IQ-TREE phylogeny


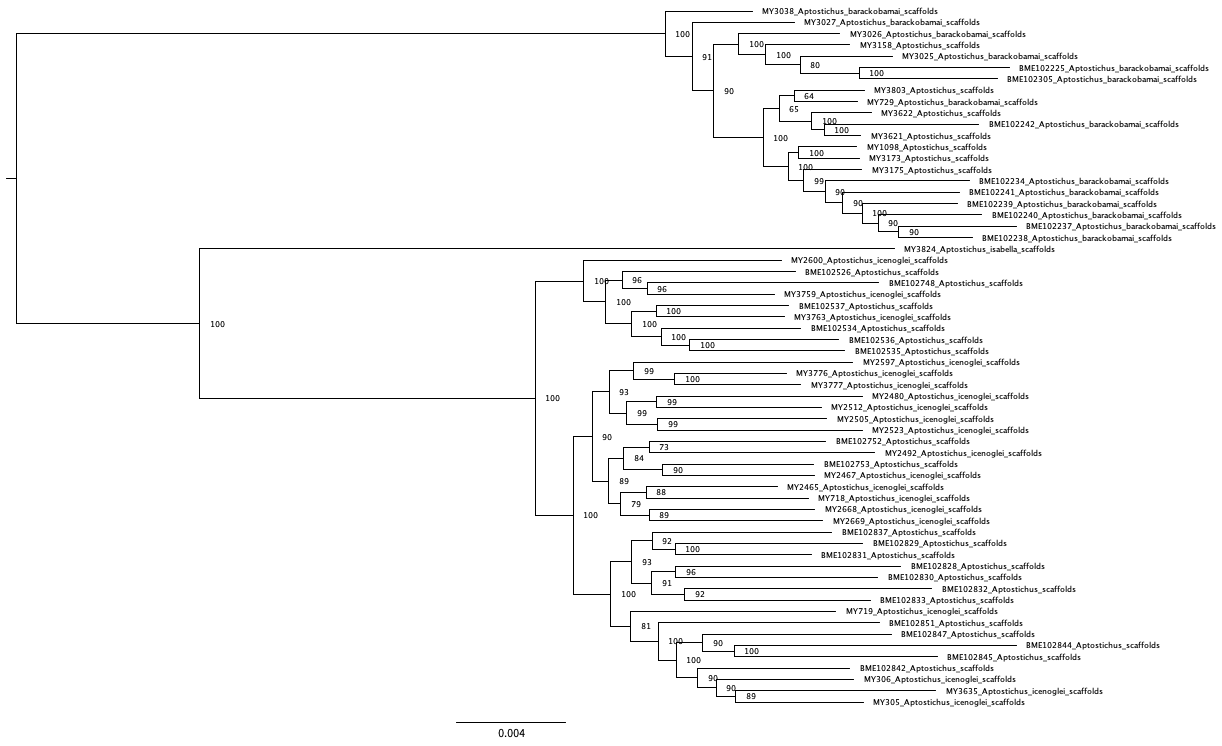


Supplemental Figure 2. 75p IQ-TREE phylogeny


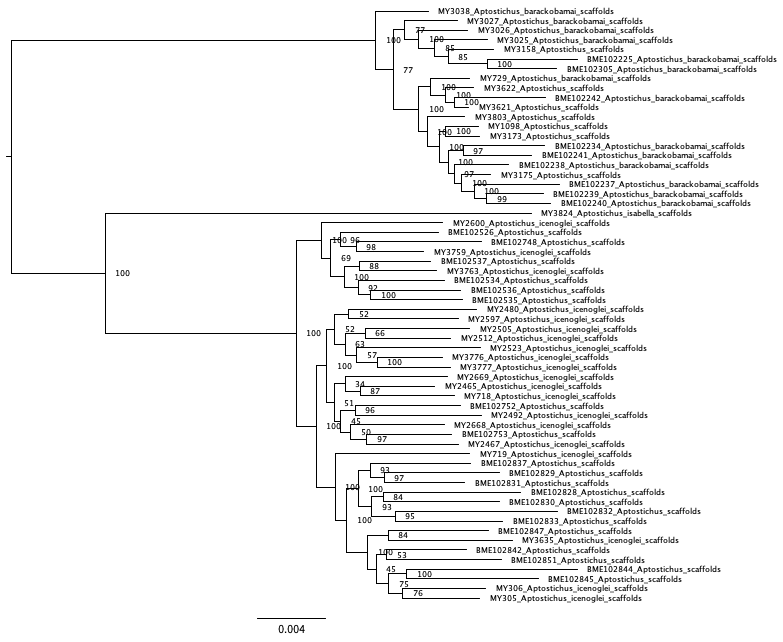


Supplemental Figure 3. 75p Astral


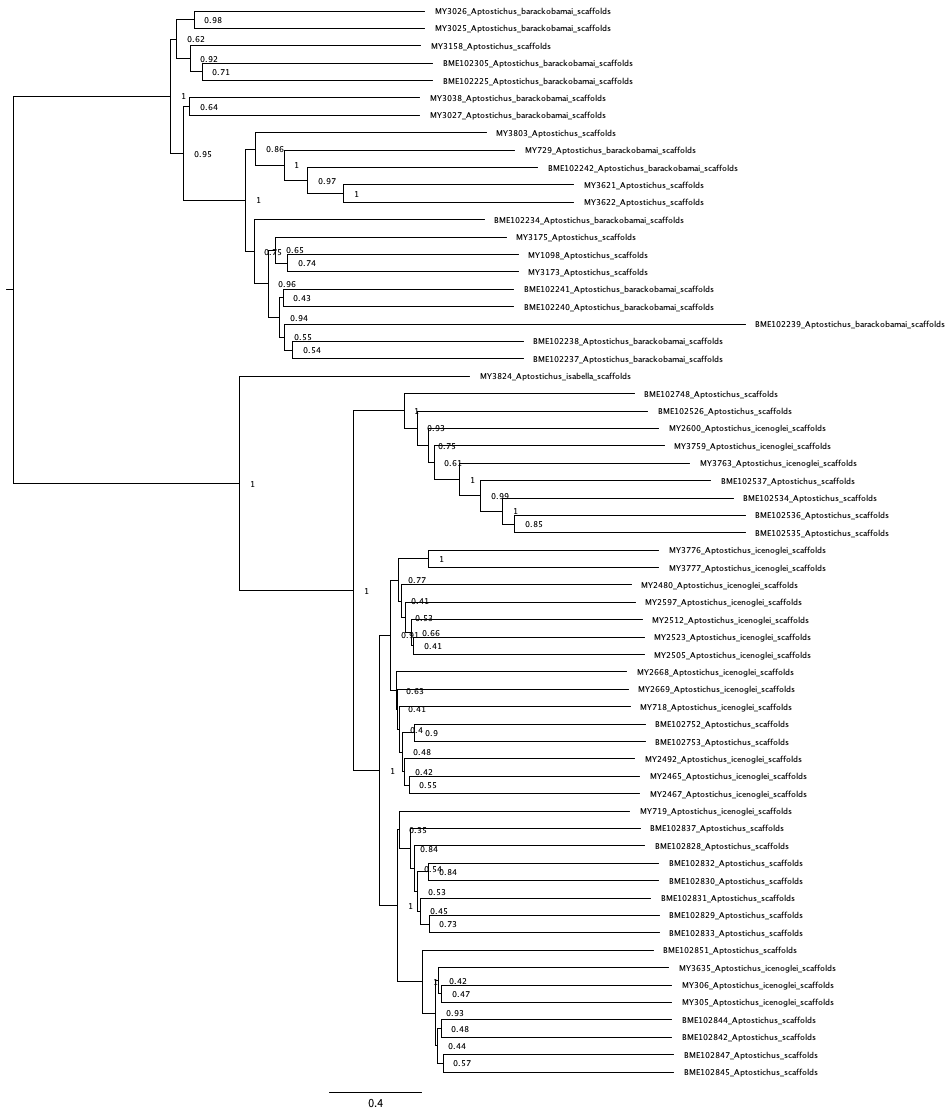


Supplemental Figure 4. 90p Astral


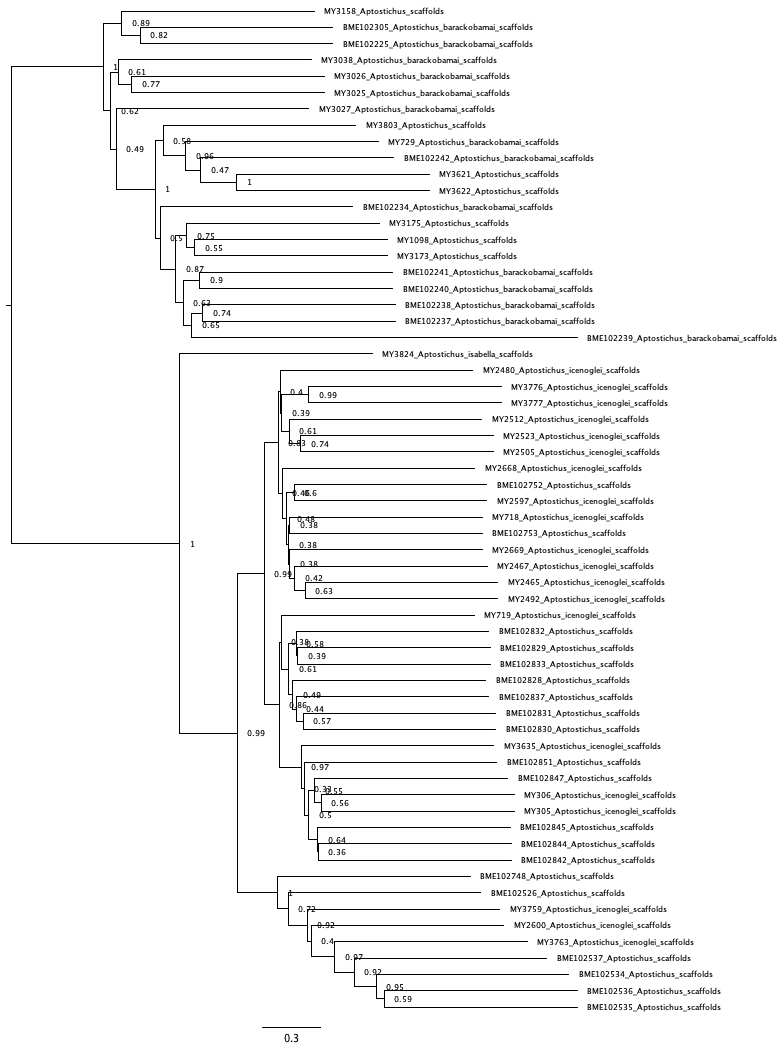


Supplemental Figure 5. 75p MSC


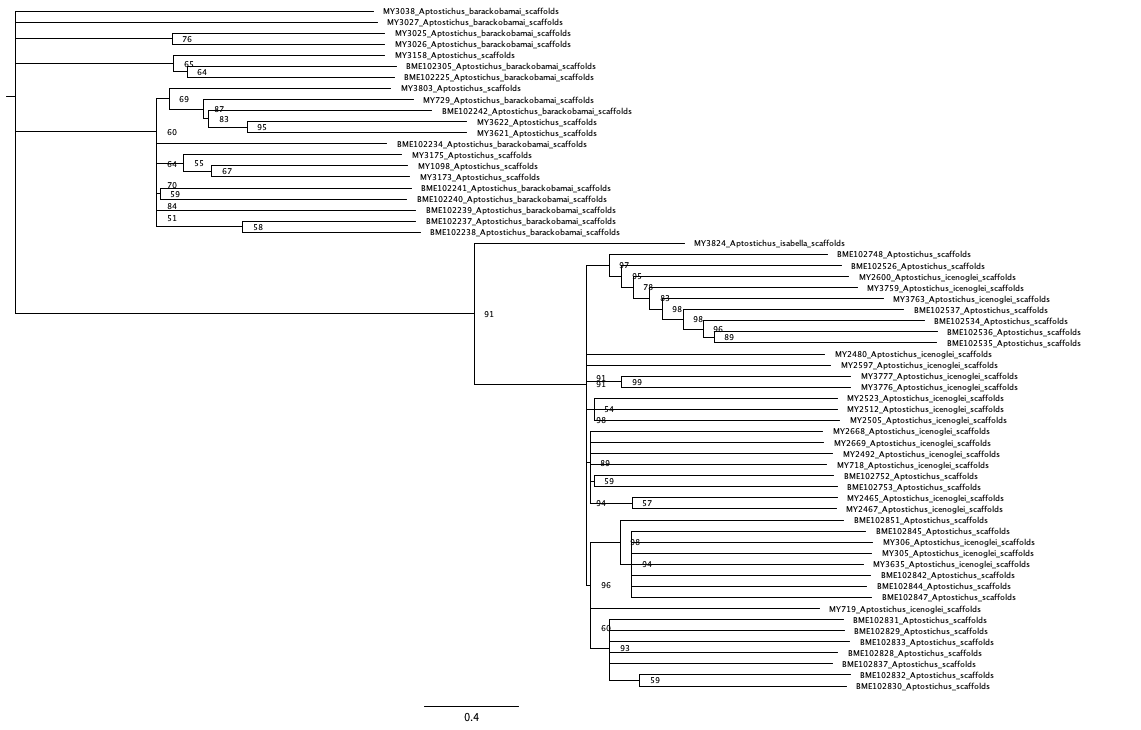


Supplemental Figure 6. 90p MSC


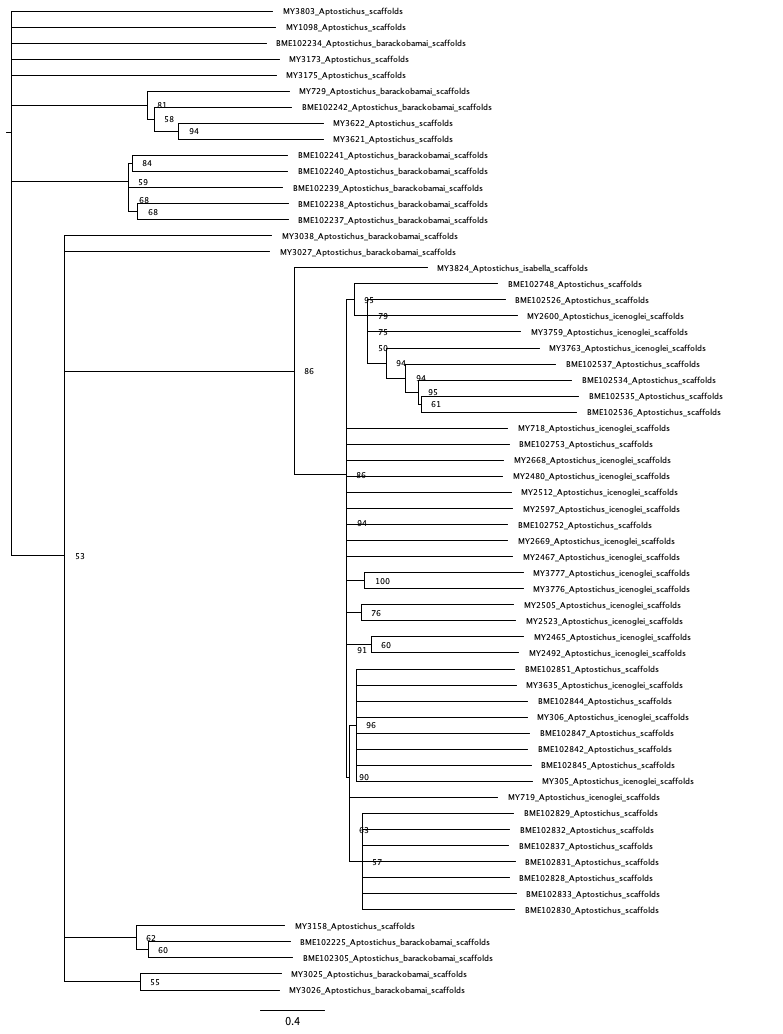


Supplemental Figure 7. Niche equivalency test results


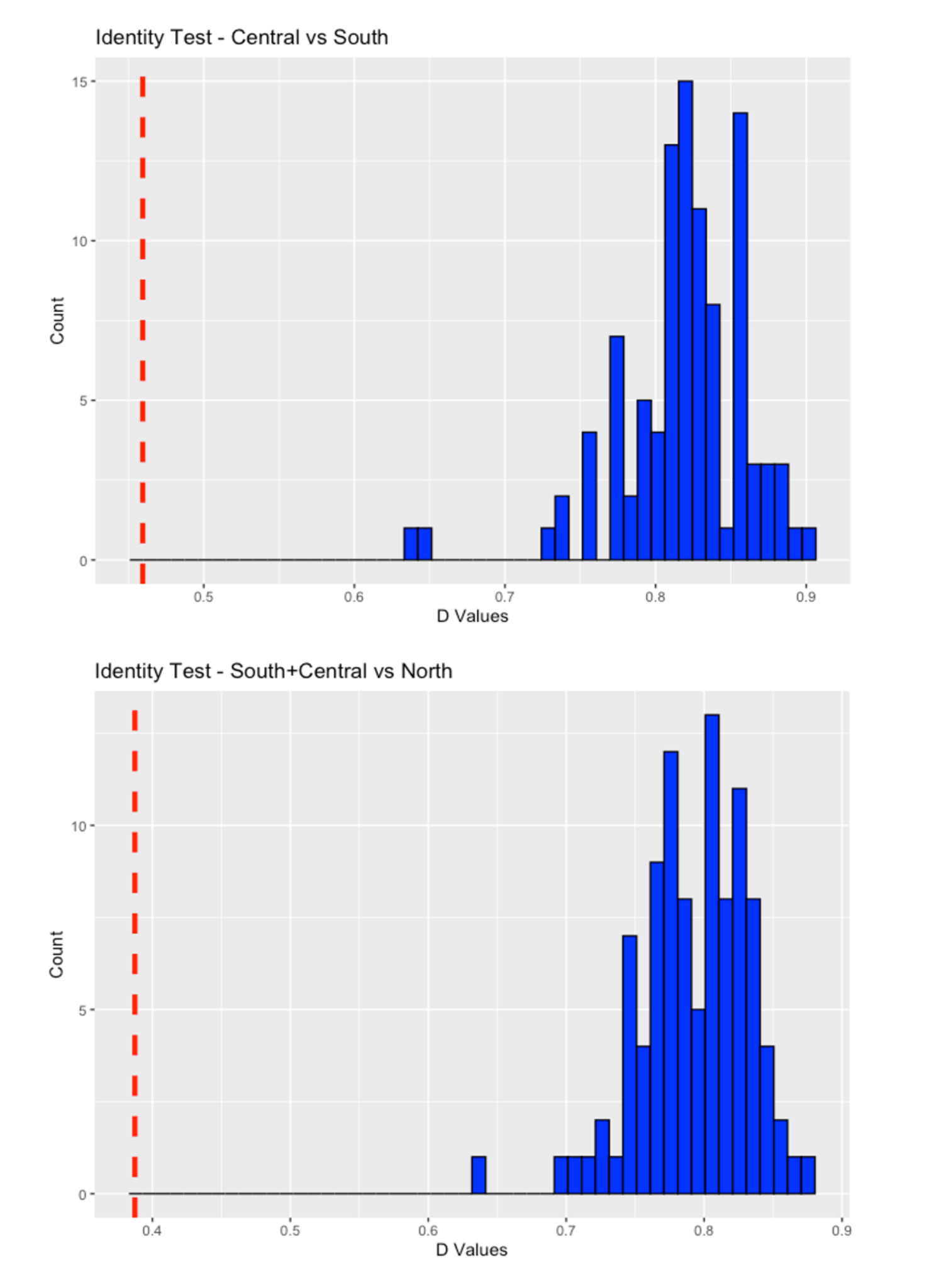


Supplemental Figure 8. Niche similarity test results for Central versus South. A) background region of minimum bounding polygon. B) background region with raster polygons where only grid cells with habitat suitability scores > 0.75 were retained. C) background region with raster polygons where only grid cells with habitat suitability scores > 0.5 were retained.


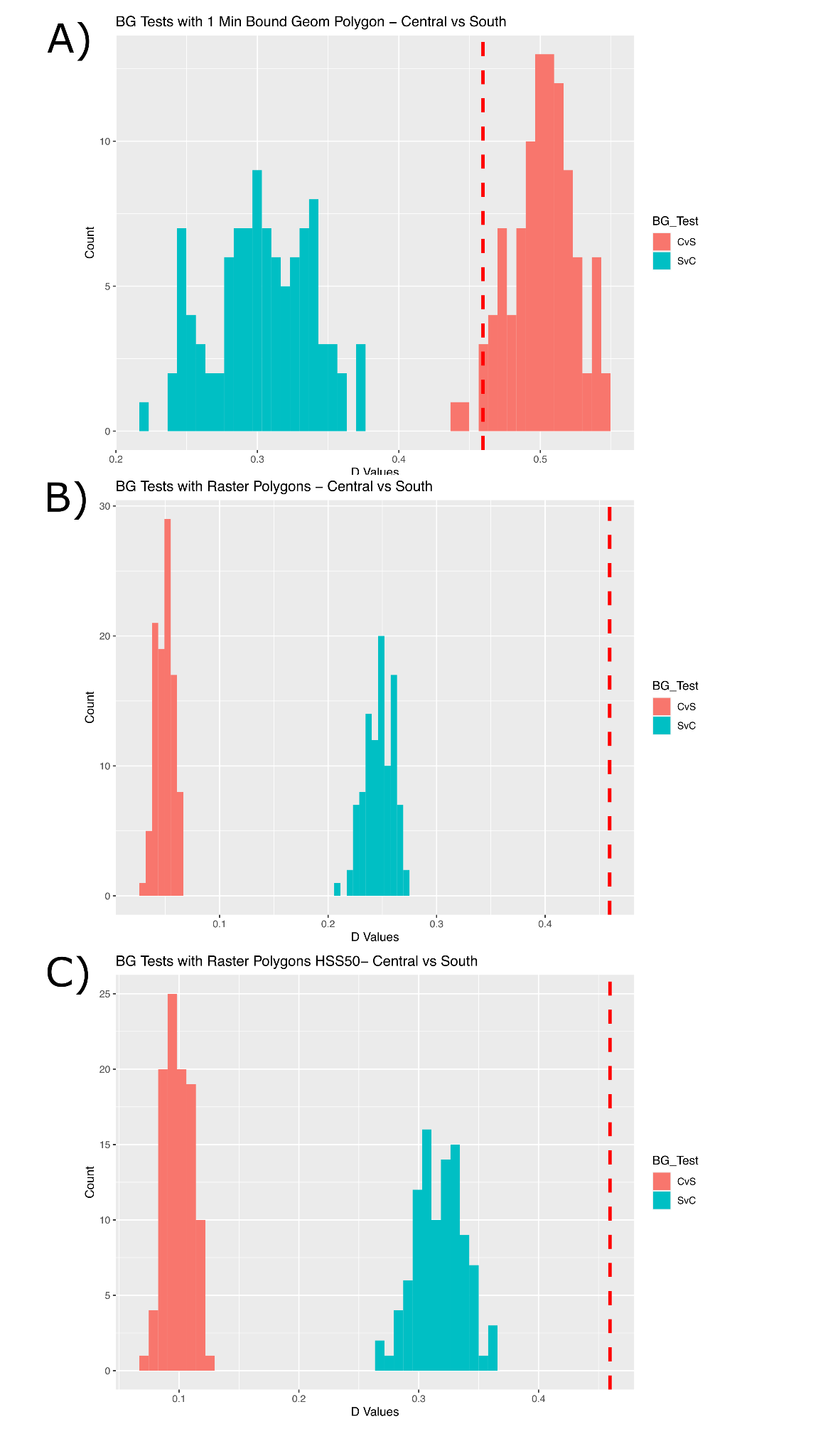


Supplemental Figure 9. Niche similarity test results for North versus Central+South. A) background region of minimum bounding polygon. B) background region with raster polygons where only grid cells with habitat suitability scores > 0.75 were retained. C) background region with raster polygons where only grid cells with habitat suitability scores > 0.5 were retained.


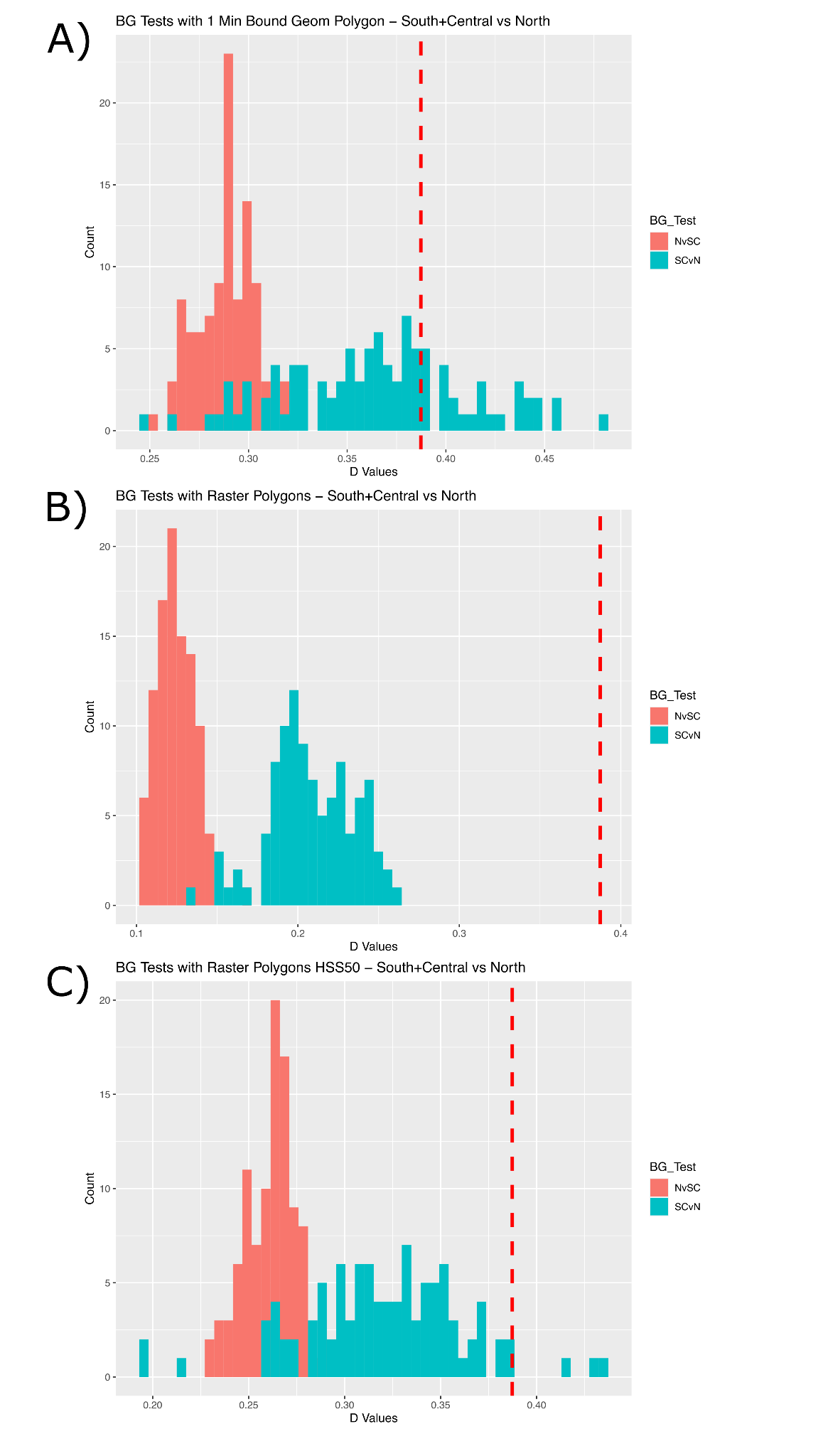


Supplemental Figure 10. Minimum Bound Geometry polygon background regions for niche similarity tests. A) polygons for the North lineage and Central+South lineage. B) polygons for the Central lineage and South lineage.


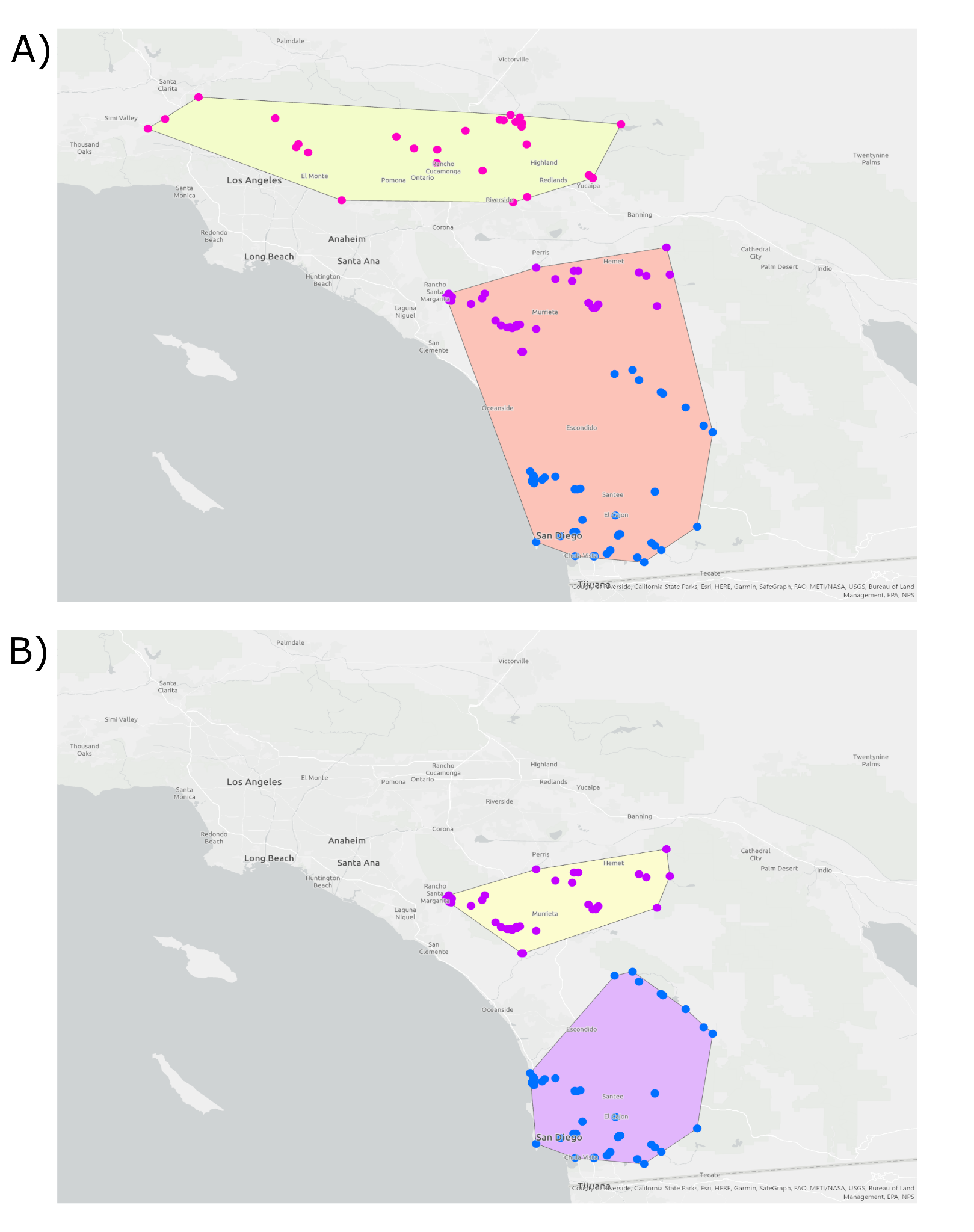


Supplemental Figure 11. Raster Polygons with HSS >0.5 and >0.75 background regions for North and Central+South niche similarity tests. A) North lineage polygons with HSS > 0.75. B) North lineage polygons with HSS > 0.5. C) Central+South lineage polygons with HSS > 0.75. D) Central+South lineage polygons with HSS > 0.5.


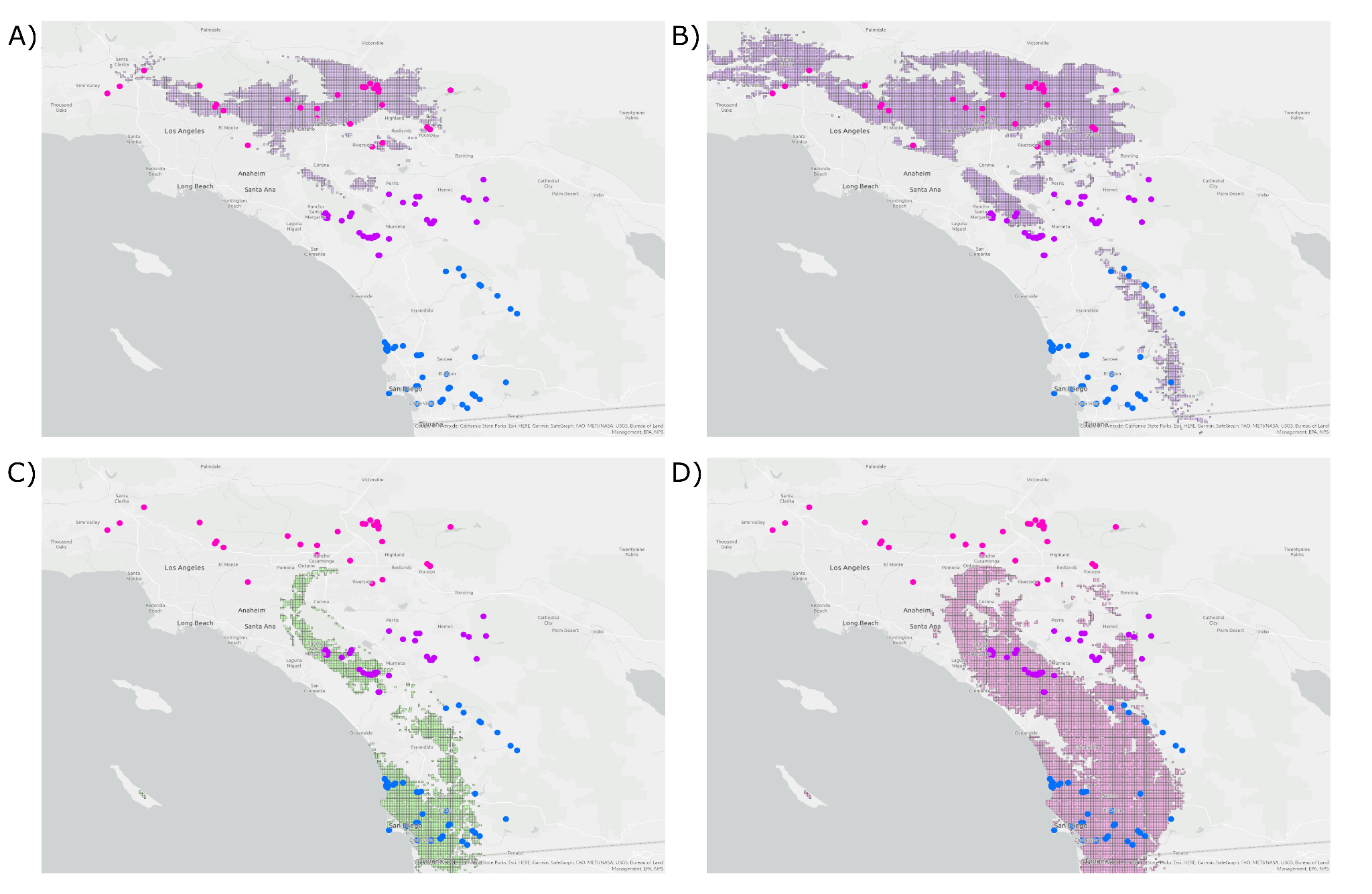


Supplemental Figure 12. Raster Polygons with HSS >0.5 and >0.75 background regions for Central and South niche similarity tests. A) Central lineage polygons with HSS > 0.75. B) Central lineage polygons with HSS > 0.5. C) South lineage polygons with HSS > 0.75. D) South lineage polygons with HSS > 0.5.


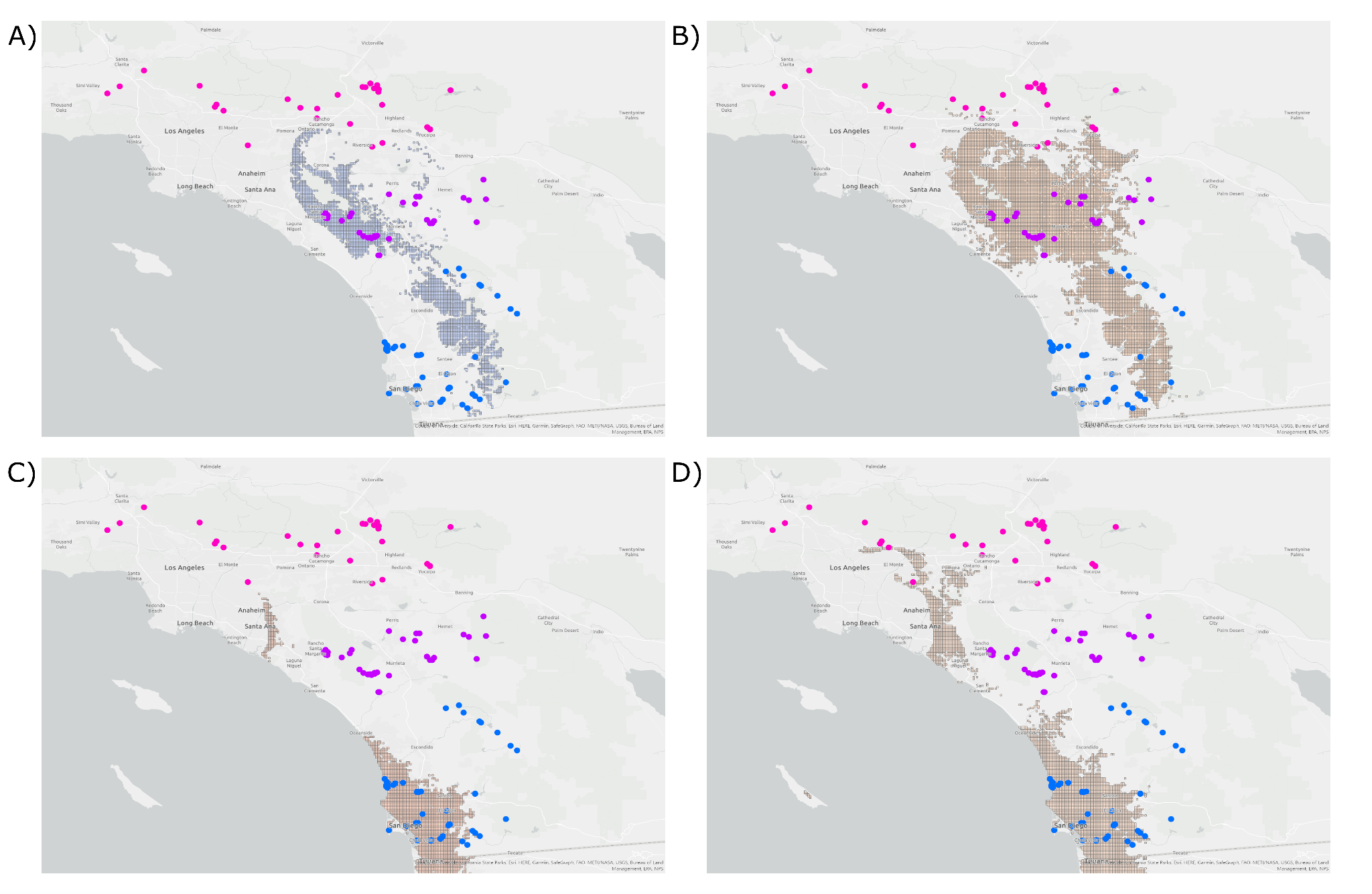


Supplemental Figure 13. 50p VAE analysis.
